# Global transcription of CRISPR loci in the human oral cavity

**DOI:** 10.1186/s12864-015-1615-0

**Published:** 2015-05-21

**Authors:** Andrew G Lum, Melissa Ly, Tasha M Santiago-Rodriguez, Mayuri Naidu, Tobias K Boehm, David T Pride

**Affiliations:** Department of Pathology, University of California, San Diego, 9500 Gilman Drive, MC 0612, La Jolla, CA 92093-0612 USA; College of Dental Medicine, Western University of Health Sciences, 309 E Second Street, Pomona, CA 91766 USA; Department of Medicine, University of California, San Diego, 9500 Gilman Drive, MC 0612, La Jolla, CA 92093-0612 USA

**Keywords:** CRISPR, Microbiome, Oral microbiome, Virome, Virus

## Abstract

**Background:**

Clustered Regularly Interspaced Short Palindromic Repeats (CRISPRs) are active in acquired resistance against bacteriophage and plasmids in a number of environments. In the human mouth, CRISPR loci evolve to counteract oral phage, but the expression of these CRISPR loci has not previously been investigated. We sequenced cDNA from CRISPR loci found in numerous different oral bacteria and compared with oral phage communities to determine whether the transcription of CRISPR loci is specifically targeted towards highly abundant phage present in the oral environment.

**Results:**

We found that of the 529,027 CRISPR spacer groups studied, 88 % could be identified in transcripts, indicating that the vast majority of CRISPR loci in the oral cavity were transcribed. There were no strong associations between CRISPR spacer repertoires and oral health status or nucleic acid type. We also compared CRISPR repertoires with oral bacteriophage communities, and found that there was no significant association between CRISPR transcripts and oral phage, regardless of the CRISPR type being evaluated. We characterized highly expressed CRISPR spacers and found that they were no more likely than other spacers to match oral phage. By reassembling the CRISPR-bearing reads into longer CRISPR loci, we found that the majority of the loci did not have spacers matching viruses found in the oral cavities of the subjects studied. For some CRISPR types, loci containing spacers matching oral phage were significantly more likely to have multiple spacers rather than a single spacer matching oral phage.

**Conclusions:**

These data suggest that the transcription of oral CRISPR loci is relatively ubiquitous and that highly expressed CRISPR spacers do not necessarily target the most abundant oral phage.

**Electronic supplementary material:**

The online version of this article (doi:10.1186/s12864-015-1615-0) contains supplementary material, which is available to authorized users.

## Background

Bacteriophage and plasmids have a significant capacity to alter the ecology of bacterial and archaeal communities [1–4]. Whether lytic phage lyse their bacterial hosts or lysogenic phage and plasmids provide them with potentially beneficial gene functions [5, 6], the presence of these mobile genetic elements are important drivers of ecosystem diversity in a number of environments [7–9]. Despite their sometimes beneficial effects upon their hosts, many bacteria and archaea have multiple mechanisms by which to counteract phage and plasmids [10–13]; thus reducing their potential to perturb microbial communities. In many bacteria and archaea, adaptive immunity has evolved, where exposures to certain phage and plasmids result in the insertion of short sequences into the host chromosome [14]. These ‘spacers’ are then utilized via nucleic acid interference to resist future encounters with the same phage/plasmids or other phage/plasmids with similar sequences [15–17]. These spacers are interspersed between palindromic repeat motifs to form the CRISPR (Clustered Regularly Interspaced Short Palindromic Repeats) locus. These loci, along with a related set of host CRISPR Associated [18] genes form the CRISPR-Cas system [19–21].

Phage generally outnumber their bacterial hosts by a 10:1 ratio in many human specimen types, and that ratio generally increases when they are attached to mucosal surfaces [22]. Relative to their bacterial hosts, there are far fewer studies characterizing the constituents, dynamics, and responses to perturbations in human phage communities [23–26]. In the human gut [27] and the human oral cavity [23], there is evidence that phage are highly persistent members of these ecosystems. This suggests that either the host organisms are incapable of utilizing CRISPRs to eradicate these viruses from the ecosystem, or that a dynamic equilibrium between host and phage has developed that may include CRISPR-mediated resistance mechanisms. Prior studies have demonstrated that phage typically coexist with CRISPR spacers that match their sequences in the human oral cavity [28, 29] and other environments [30]. Local phage populations respond to the presence of matching CRISPR spacers in a number of environments [31, 32], but these dynamics have not been thoroughly explored in complex human specimens types. In human periodontal disease, phage communities are noted to be significantly altered compared to periodontally healthy subjects [33]. Whether there may be a role for CRISPRs in the cellular microbiota in helping to shape these differences amongst the periodontal microbiota has not previously been explored. There are substantial repertoires of CRISPR spacers in human saliva [11], dental plaque [34], skin [35], and the GI tract [36]; however, the expression of these CRISPR spacers has yet to be examined.

Studies of the distribution of CRISPR spacers in human subjects have revealed that despite a fair number shared between individuals, they are highly individual-specific [35]. In addition to subject-specificity, CRISPR spacers vary according to biogeographic sites sampled [28, 34]. There are numerous shared spacers between dental plaque and saliva in the oral cavities of individual subjects; however, each site has its own distinct biogeography. Many of the CRISPR spacers found on different body surfaces are highly conserved over time [35], as evidenced by the large proportion of spacers conserved longitudinally on human skin and in saliva. Much of the conservation of CRISPR spacers likely is due to the persistence of the bacteria harboring them on these body surfaces. Despite having significant differences in their bacterial ecology, many CRISPR spacers in the human mouth are identical to those found on human skin [35], which suggests that both body sites are exposed to similar viruses; however, some CRISPRs on both body surfaces are probably acquired through either vertical or horizontal transmission. Individuals living together share a significant proportion of their CRISPR spacers when compared to control individuals from different living environments, which likely reflects a combination of shared viral exposures and shared bacterial biota within a household [29]. We hypothesize that many of these CRISPR loci are actively transcribed and may be involved in acquired resistance against oral bacteriophage; however, the activity of CRISPR loci has not previously been evaluated on a global scale on human body surfaces.

CRISPR loci are transcribed as a single, long RNA precursor that is processed to generate CRISPR RNAs (crRNAs). Transcription is generally unidirectional, and initiates at the end of the locus that contains the leader sequence [17]. Cas proteins are necessary for the processing of the long CRISPR transcript, but the regulation of Cas genes is likely to differ according to bacterial species or strain [37, 38]. CRISPR-Cas-mediated resistance then generally proceeds in three stages in the CRISPR-Cas systems described thus far [39]. In the first stage, Cas proteins recognize and cleave exogenous sequences and incorporate them next to *cas* genes; the second stage involves the transcription of crRNAs, and the third stage involves the interference mechanism [21]. Type II CRISPR-Cas systems have been well characterized in *Streptococcus thermophilus* [40], and consist of *cas1*, *cas2*, *cas9* and either *csn2* or *cas4* [39]. Several studies of CRISPRs in the human oral cavity have characterized Type II CRISPR-Cas systems, alternatively known as SGI and SGII CRISPRs based on their repeat motif sequences in streptococcal species [11, 28, 29, 34, 35]. Cas1 and Cas2 are universal among the CRISPR-Cas systems and the others are unique to the Type II CRISPR-Cas system [41, 42]. Cas9 degrades foreign DNA and functions in crRNA biogenesis [43], and Csn2 and Cas4 are important for new spacer acquisition in Type II-A, and type II-B CRISPR-Cas systems, respectively [44, 45]. While CRISPR-Cas transcription mechanisms have been characterized in environmental settings and various bacteria/bacteriophage models [30, 46, 47], transcription of CRISPR loci has not been evaluated in human ecosystems. Many bacteria and archaea in these environments may also harbor CRISPR loci that are inactive [19, 48–53], which may occur at the level of transcription of the CRISPR locus or the expression of Cas genes.

Here, we sought to characterize CRISPR loci at the community level in human saliva to decipher whether CRISPR locus transcription is targeted towards highly abundant oral phage. Because we amplify and sequence spacers belonging to CRISPR loci based on their repeat motifs, we can simultaneously characterize hundreds of different CRISPR loci belonging to different oral bacteria [29]. In our prior studies of oral CRISPRs, we have found that the proportion of CRISPR spacers that matched oral phage was relatively low. Due to the high numbers of CRISPR loci that we can identify and the low proportion of matching oral phage, we hypothesized that the proportion of CRISPR loci identified in the oral cavity that are expressed as transcripts would be low. The goals of this study were to compare the CRISPR repertoires found in genomic DNA with those present in mRNA in a cohort of human subjects, examine whether biases exist in CRISPR repertoires that may be characteristic of oral health status, identify whether many CRISPR loci are natively transcribed, develop methods for assembly of CRISPR loci from short sequence reads, identify highly expressed CRISPR spacers, and to determine whether the transcription of CRISPR loci may be regulated by the presence of highly abundant oral viruses.

## Results

### CRISPR spacer sequencing

We recruited 16 human subjects and sampled their saliva. Nine of the subjects were in good overall periodontal health and 7 had significant periodontal disease. We amplified and sequenced CRISPR spacers by utilizing their direct repeat motifs as targets for the primer sequences. The benefit of such a technique is that we can amplify CRISPR spacer sequences from a wide array of different bacterial species that share the same repeat motifs, while the primary limitation is that we cannot ascribe the spacer sequences to any given bacterial species or strain. We sequenced SGI and SGII CRISPR spacers (both are Type II CRISPR-Cas systems) that have previously been identified primarily in various species of *Streptococcus*, GHI spacers identified in *Gemella haemolysans*, and VSI spacers identified in *Veillonella* species [28, 35]. GHI and VSI CRISPR repeat motifs have not previously been assigned to CRISPR-Cas system types. We previously have shown that there are robust repertoires of each of these CRISPR spacer types in a larger group of human subjects [29]. Each spacer type likely is present in multiple different oral bacteria species. From the DNA of each subject, we sequenced 224,896 SGI CRISPR spacers (mean of 14,056 per subject), 282,903 SGII spacers (mean of 17,681 per subject), 397,687 GHI spacers (mean of 24,855 per subject), and 463,368 VSI spacers (mean of 28,961 per subject) (Additional file 1: Table S1). We also sequenced CRISPR spacers from the mRNA of each subject to determine whether CRISPR spacers present in each subject were transcribed. From the cDNA, we sequenced 219,113 spacers SGI spacers (mean of 13,695 per subject), 195,316 SGII spacers (mean of 12,207 per subject), 352,071 GHI spacers (mean of 22,004 per subject), and 303,764 VSI spacers (mean of 18,985 per subject).

### Spacer binning and estimated coverage

We binned the spacer sequences according to our previously described protocols based on their trinucleotide content to correct for potential sequencing errors as well as to group highly similar spacer sequences [29]. Similar to data utilizing a different group of subjects, the vast majority of CRISPR spacers from each spacer type was identical and did not necessitate grouping according to trinucleotide content. The estimated polymorphism rate among the spacers was 0.001 % for SGI spacers, 0.002 % for SGII spacers, 0.003 % for GHI spacers, and 0.003 % for VSI spacers (Additional file 2: Figure S1). We next performed rarefaction analysis on the resulting spacer groups to get an estimate of the spacer richness in each subject or group of subjects and to determine whether the majority of the spacer sequences had been sampled. We found that for most subjects and CRISPR spacer types the curves reached asymptote, indicating that further sequencing would not have revealed many new spacer sequences (Additional file 2: Figure S2, Panels A-D). For SGII spacers, there generally was higher spacer richness, and fewer of the curves reached asymptote. There was no substantial association between oral health status and CRISPR spacer richness for SGI spacers (Panel A), SGII spacers (Panel B), or VSI spacers (Panel D); however, there was a trend towards lower spacer richness for GHI spacers in subjects with periodontal disease (Panel C). Nucleic acid type was not associated with CRISPR spacer richness, as regardless of CRISPR spacer type, curves were similar in each subject for those spacers identified from DNA and those identified from cDNA (Panels A-D).

### CRISPR spacer distribution by subject and oral health status

**Fig. 1:**
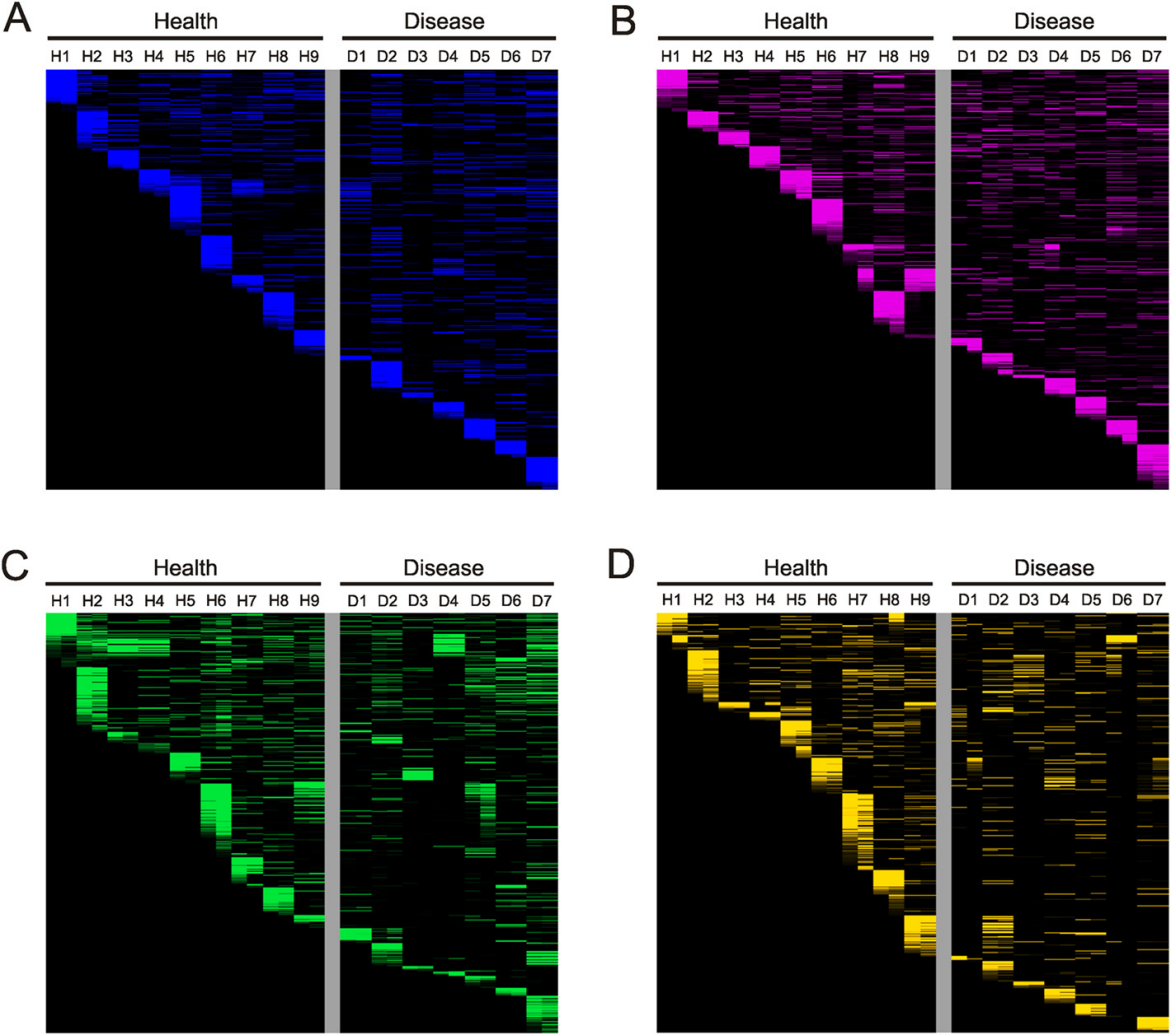
Heatmaps of CRISPR spacer groups in all subjects and nucleic acid types. Each row represents a unique spacer group and the columns are labeled by each individual subject. For each subject, CRISPR spacers derived from genomic DNA are located on the left and CRISPR spacers derived from cDNA are located on the right. Panel **A**—SGI CRISPR spacers, Panel **B**—SGII CRISPR spacers, Panel **C**—GHI CRISPR spacers, and Panel **D**—VSI CRISPR spacers

**Fig. 2:**
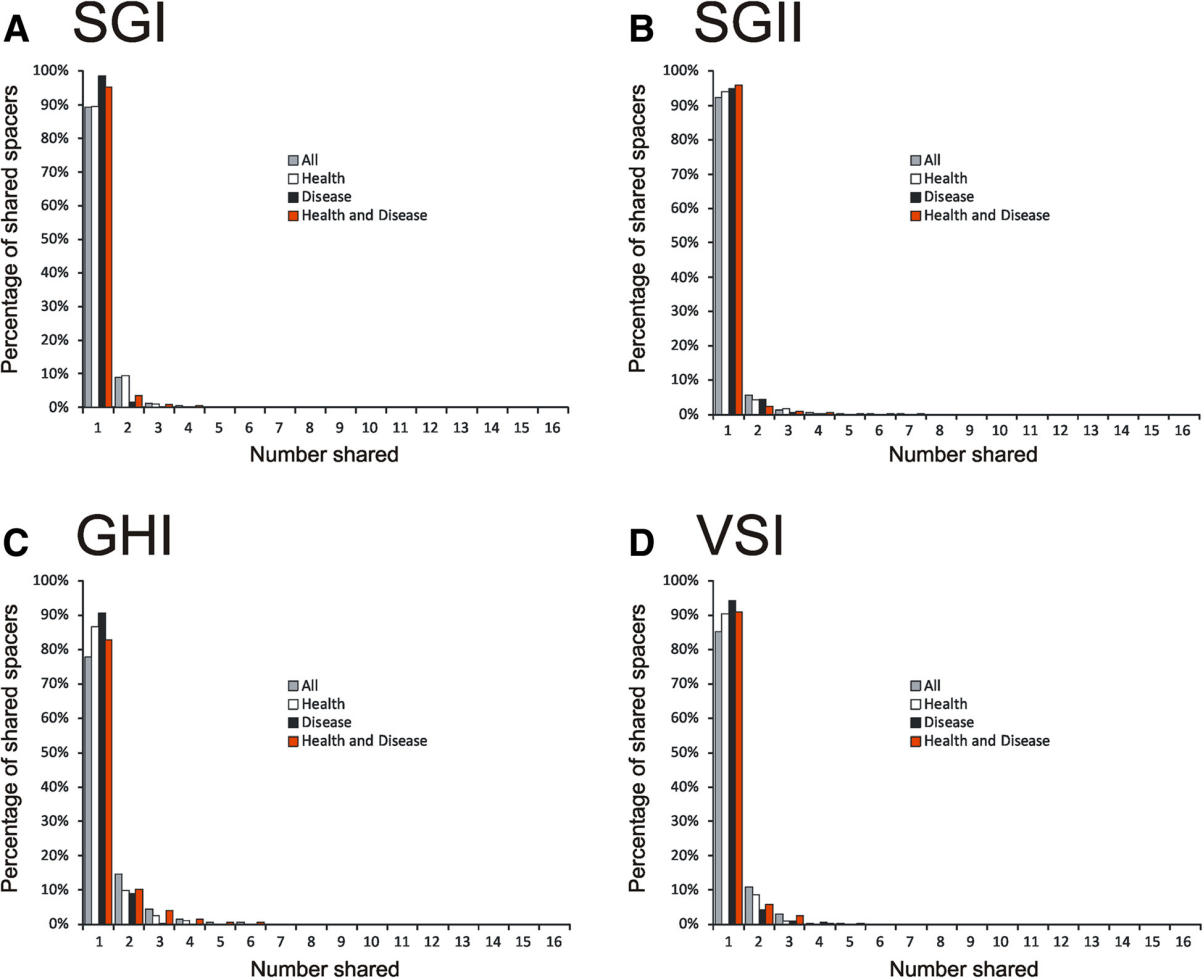
Bar graphs representing the percentages of shared spacers across all subjects and spacer types. Spacers shared when all subjects are compared are shown in gray, when only subjects with periodontal health are compared are shown in white, when only subjects with periodontal disease are compared are shown in black, and for comparisons between subjects with periodontal health and disease are shown in red. The y-axis represents the percentage of spacers shared, and the x-axis represents the number of different subjects sharing spacers. The bars representing ‘1’ are unique to individual subjects, and ‘2’ represents spacers shared by only 2 different subjects, and so on. Few spacers across all spacer types are shared in more than 2 different subjects. Panel **A**—SGI CRISPR spacers, Panel **B**—SGII CRISPR spacers, Panel **C**—GHI CRISPR spacers, and Panel **D**—VSI CRISPR spacers

We next characterized the distribution of CRISPR spacers amongst each subject and nucleic acid type to determine whether there were CRISPR spacers more likely to be present or transcribed by oral health status. The majority of the SGI (Fig. 1, Panel A), SGII (Panel B), GHI (Panel C), and VSI (Panel D) CRISPR spacers appear to be subject specific rather than specific to oral health status when examining CRISPR spacer distribution by heatmap. We quantified the proportions of CRISPR spacers shared amongst subjects with periodontal health or disease and found that 13.5 % of SGI spacers, 12.8 % of SGII spacers, 21.1 % of GHI spacers, and 12.5 % of VSI spacers were shared between subjects with relative periodontal health (Additional file 1: Table S2); none of these values were significantly different from the proportion of spacers shared in periodontal disease or the proportion shared between subject groups. The vast majority of the spacers in each subject were individual specific, and those 10-15 % that were shared between different subjects were largely shared with only 1 other subject for all spacer types (Fig. 2, Panels A-D). There were few spacers that were shared between 2 or more subjects and no specific patterns observed of spacer sharing between subjects with relative periodontal health and disease. When measuring the relative proportion of CRISPR spacers shared within a subject versus those shared with other subjects, we found that in nearly all subjects and CRISPR spacer types, the CRISPR spacer repertoires were significantly (p < 0.05) subject specific (Additional file 1: Table S3).

### CRISPR spacer transcripts

**Fig. 3:**
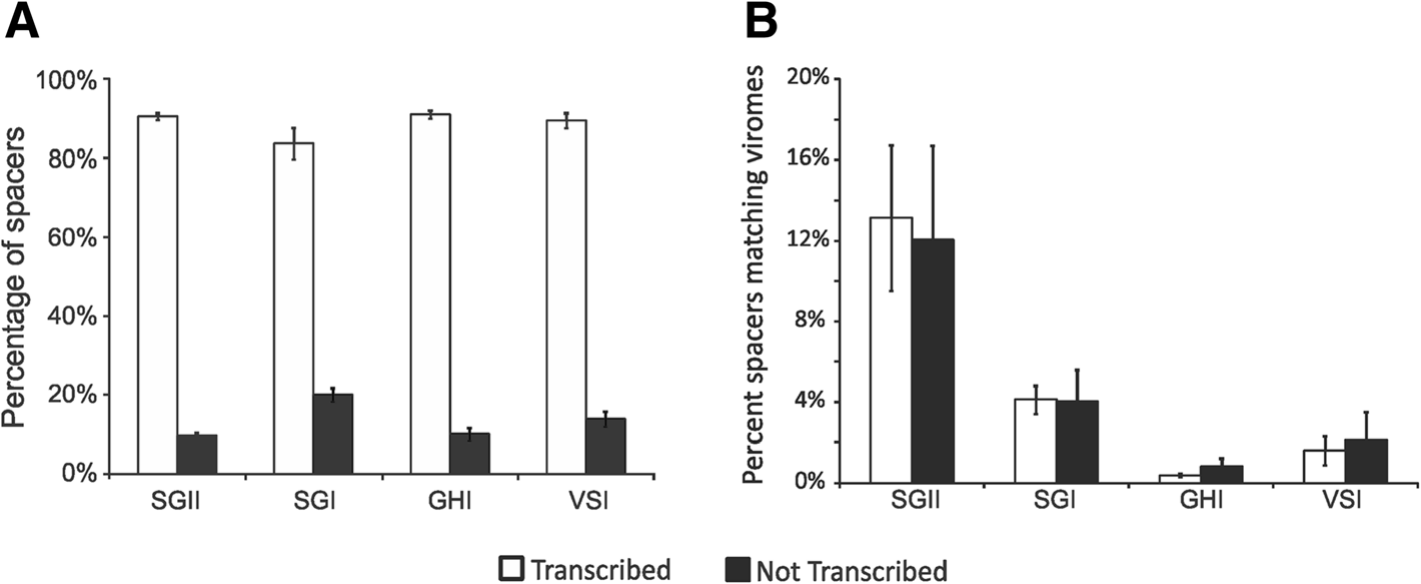
Percentage (±standard deviation) of spacers from each CRISPR spacer type that are transcribed for all subjects (Panel **A**), and the percentage of spacers that match virome reads (Panel **B**). Transcribed spacers are shown in white and those not identified in transcripts are shown in black

Many of the CRISPR spacers identified in each subject appeared to also be present in transcripts for most subjects and CRISPR types (Fig. 1). We quantified the proportion of CRISPR spacers in each subject that were present in the DNA, but also were found in transcripts (Fig. 3, Panel A). The vast majority of all CRISPR spacers identified also were transcribed, with 95.4 ± 11.7 % of SGII spacers found in both the DNA and RNA fractions, while 4.6 ± 2.5 % were found in the DNA fractions alone. Similar results were identified for SGI (88.4 ± 8.2 % present in both), GHI (86.7 ± 7.4 % present in both), and VSI (79.6 ± 11.5 % present in both) spacers, indicating that the vast majority of the CRISPR spacers were transcribed. The proportions of spacers shared in the DNA and cDNA fractions were significantly subject specific (p < 0.05) in nearly all subjects and CRISPR types (Additional file 2: Figure S3). There was no association between the CRISPR spacer repertoires of different subjects based on whether they were transcribed or not, as evidenced by the 8.9 % of SGI spacers, 9.9 % of SGII spacers, 15.4 % of GHI spacers, and 10.4 % of VSI spacers that were shared between different subjects in the cDNA fractions. These relative proportions were similar to those shared in the DNA fractions between different subjects, and none of the differences were significant based on nucleic acid type (Additional file 1: Table S2).

### Matches to virome reads

We previously sequenced the viromes from the saliva of each subject involved in this study [33]. We compared the relative proportions of the CRISPR spacer repertoires of each CRISPR spacer type that matched virome reads to determine whether spacers that are transcribed may be targeted towards the most abundant viruses present in the oral cavity. We measured the proportions of CRISPR spacers matching virome reads for those that were transcribed (found in DNA and RNA fractions), and those that were not transcribed (found in the DNA fraction only). Because the proportion of spacers matching phage within the same subject was relatively low in this study and in prior studies of oral CRISPR/Phage interactions [28, 29, 34, 35], we pooled the virome reads to identify matches to phage known to be present in the oral cavity of our cohort. There were no significant trends or differences between transcribed spacers and those spacers not transcribed in their relative proportions that matched virome reads (Fig. 3, Panel B). For example, 13 % of the transcribed SGII CRISPR spacers matched virome reads, while 12 % of the spacers that were not transcribed matched virome reads; a difference that was not statistically significant. We also tested whether there was an association between the spacers that matched the virome reads based on the oral health status of each subject. We observed mostly subject specific patterns of spacers that matched virome reads for SGI and SGII spacers (Additional file 2: Figure S4), and no associations were observed based on periodontal health status for any of the spacer types evaluated (Additional file 2: Figure S5).

### Identification of highly expressed spacers

**Fig. 4:**
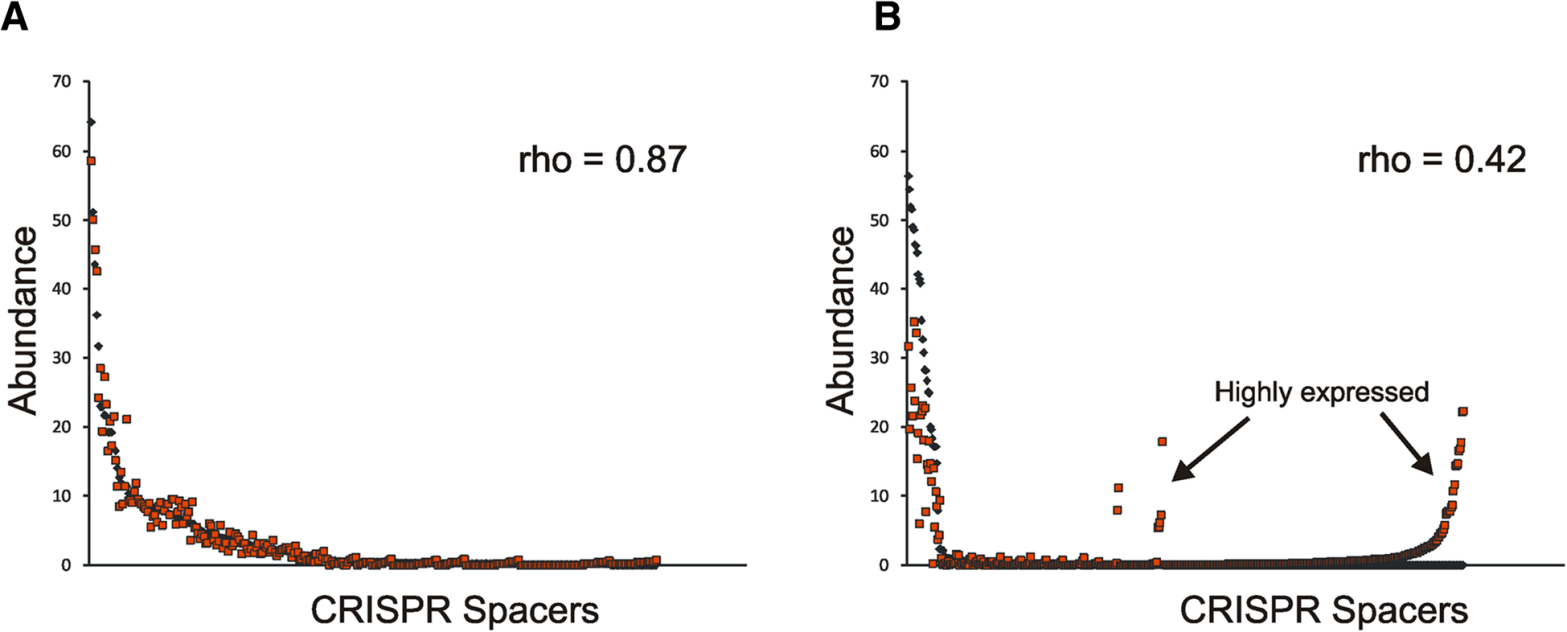
Plots of PPTS (Percentage Per Thousand Spacers) values from the DNA fractions (black diamonds) and the cDNA fractions (red boxes). The PPTS values are shown on the y-axis and the individual spacers are shown on the x-axis. Each box or diamond represents a different individual spacer. Spearman’s rho values are represented above the curves. PPTS values that differ significantly between DNA and cDNA fractions are shown by the arrows. Panel **A** represents SGI spacers for subject H1 and Panel **B** represents SGI spacers for subject H7

**Fig. 5:**
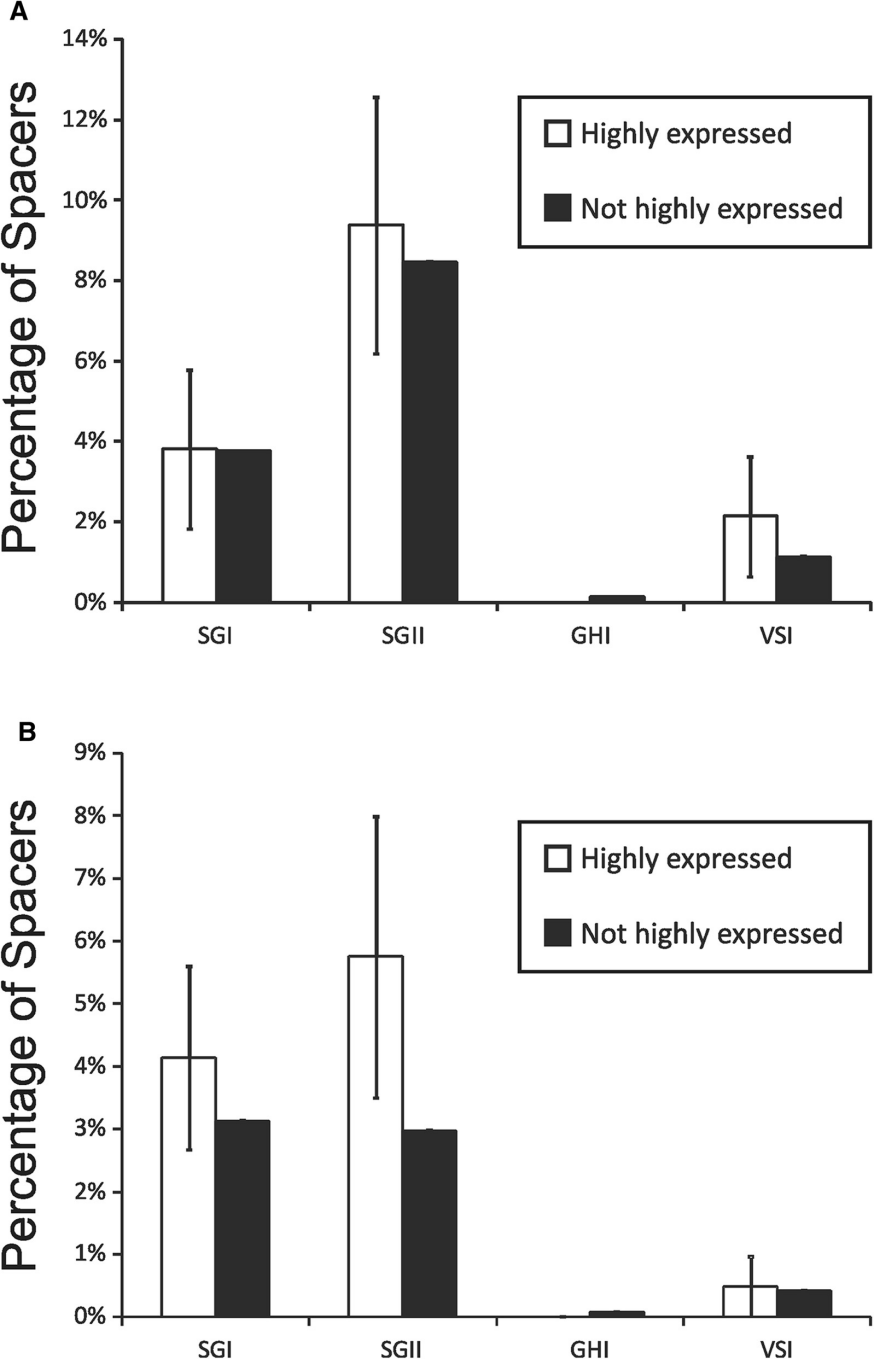
Bar graphs of the percentages (±standard deviation) of highly expressed spacers that match virome reads (Panel **A**) or that have homologues in the NCBI NR database (Panel **B**). Highly expressed spacers are represented by white bars and all other spacers are represented by black bars

We normalized spacer values according to their Percentage Per Thousand Spacers (PPTS) so that we could directly compare their proportions across DNA and cDNA. The relative abundances of spacers in the DNA fraction were used as baseline expression values and compared to relative abundances in cDNA to determine whether some spacers were highly expressed. We then utilized a rank correlation method based on the PPTS values to characterize how well the relationship between the baseline expression and the expression values in the cDNA were correlated. For most subjects and spacer types, there was a substantial correlation between the baseline expression values and those found in the cDNA (Fig. 4, Panel A and Additional file 2: Figures S6, S7, S8 and S9), which suggested that relatively few spacers had significantly altered expression. We calculated Spearman’s rho values to describe the degree of correlation between the expression values. In general, values ranging from 0.6 to 1.0 are considered strong correlations, values of 0.4 to 0.6 are considered moderate, and values below 0.4 are considered weak. Most values exceeded 0.6 for all spacer types, while only a couple of values indicated weak correlations (Table 1). For example, SGI spacers for subject H1 had a high degree of correlation with a rho value of 0.87 (Fig. 4, Panel A), while there were quite a few SGI spacers in subject H7 that appeared to be highly expressed with a rho value of 0.42 (Panel B). To decipher whether those spacers that appeared to be highly expressed were actually highly expressed, we calculated residual values between the PPTS in the DNA and the cDNA and defined those spacers with residual values greater than 3x the baseline expression as highly expressed. Spacers that met this definition of high expression represented approximately 9-11 % of the spacers repertoires evaluated across all subjects and CRISPR types (Additional file 2: Figure S10). We next evaluated whether those spacers that were highly expressed were more likely to match oral phage from the subjects studied. There were no significant differences identified in the proportions of spacers matching virome reads regardless of whether they were highly expressed or not (Fig. 5, Panel A). There was a trend towards more of the highly expressed SGI and SGII spacers matching phage present in the NCBI NR database, but these results were not statistically significant (Panel B).

**Table 1:** Spearman rho values for all DNA/cDNA comparisons

	DNA vs cDNA CRISPR spacer comparisons			
Subject	SGI	SGII	GHI	VSI
H1	0.87	0.77	0.85	0.53
H2	0.89	0.65	0.55	0.70
H3	0.71	0.87	0.91	0.83
H4	0.90	0.87	0.88	0.82
H5	0.70	0.87	0.77	0.77
H6	0.85	0.85	0.81	0.86
H7	**0.42** ^**a**^	0.79	0.67	0.53
H8	0.85	0.85	0.89	0.61
H9	0.84	0.88	0.84	0.72
D1	0.57	0.88	0.82	**0.46**
D2	0.61	0.93	0.84	0.84
D3	0.56	0.70	0.87	0.82
D4	0.53	0.87	0.58	0.84
D5	0.87	0.80	0.57	0.75
D6	0.80	0.86	0.93	0.59
D7	0.80	0.83	0.83	0.79

^a^rho values <0.5 are indicated in bold

### CRISPR locus assembly technique

**Fig. 6:**
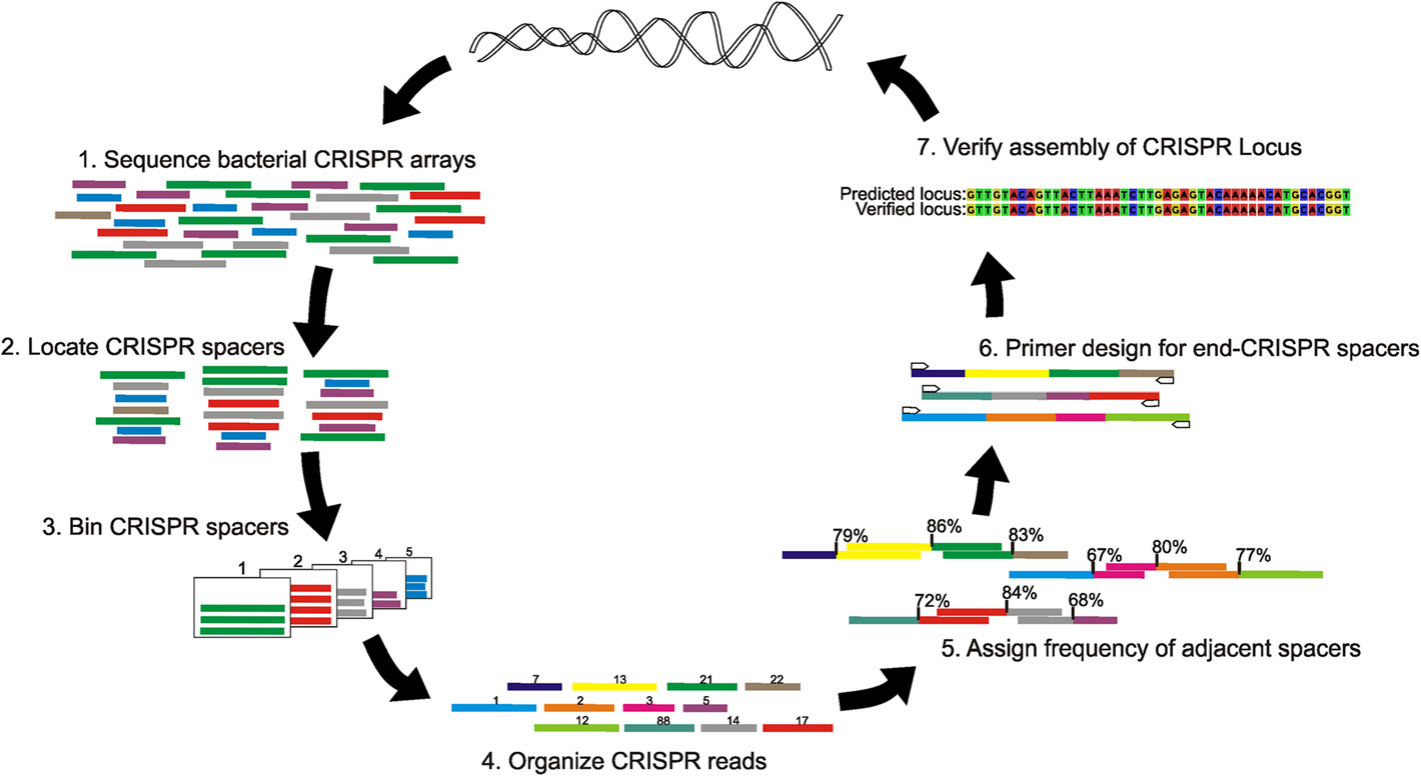
Diagram of workflow to reconstruct CRISPR loci from short sequence reads

Because of the significant number of SGI and SGII CRISPR spacers that matched virome reads from the subjects (Fig. 3, Panel B), we developed a method to assemble CRISPR loci from each subject to determine whether certain loci had multiple spacers that matched oral phage. To assemble CRISPR loci, we re-sequenced all the SGI and SGII CRISPR spacers from each subject using longer read technology, where the average read length was approximately 200 bp and contained multiple spacers compared to the shorter reads (average length of 100 bp) used to characterize the CRISPR spacer repertoires. From the DNA of each subject, we sequenced 264,749 SGI spacers (mean of 16,547 per subject) and 310,953 SGII CRISPR spacers (mean of 19,435 per subject) (Additional file 1: Table S4). From the cDNA, we sequenced 375,625 SGI spacers (mean of 23,477 per subject) and 327,748 SGII CRISPR spacers (mean of 20,484 per subject). We binned the spacers from each sequence read according to their trinucleotide content to account for the potential for sequencing errors, and assigned each spacer a unique identifier (Fig. 6). We then compared the reads within each subject by nucleic acid type to reassemble CRISPR loci based on their overlapping adjacent spacers. Only spacer combinations that were present in 60 % of the reads were utilized to reconstruct CRISPR loci, while reads that had similar CRISPR spacers within a subject with their order altered were discarded, as they may represent lesser abundant CRISPR loci or could occur from PCR amplification artifacts. We validated that the technique is capable of reconstructing CRISPR loci by PCR amplifying and sequencing the entire reconstructed locus using traditional Sanger sequencing (Additional file 2: Figures S11 and S12).

### Analysis of CRISPR loci

We binned the SGI spacers from DNA into 3,045 spacer groups and the SGII spacers into 3,268 spacer groups. From the cDNA, we binned the SGI spacers into 3,200 spacer groups and the SGII spacers into 3,219 spacer groups. We performed rarefaction analysis on each subject and nucleic acid type for SGI (Additional file 2: Figure S13, Panel A) and SGII (Panel B) to determine whether the CRISPR spacers had been thoroughly sampled. In each case, the rarefaction curves reached asymptote, indicating that further sequencing would not have identified many more CRISPR spacers in each subject. The relative spacer richness estimates were lower for the longer reads than for the short-read sequencing (Additional file 2: Figure S2) because short reads that likely contained orphan spacers were not included in this analysis.

After assembly, we quantified the metrics of the resulting CRISPR loci. From the cDNA for SGI CRISPRs, the mean number of loci was 23.6 per subject with an average of 5.8 spacers per locus (Additional file 1: Table S5). Similar results were found in the DNA for SGI CRISPRs with a mean of 21.7 loci per subject with an average of 5.8 spacers per locus. For SGII CRISPRs, there were 20.3 loci per subject with a mean of 7.3 spacers per locus from the cDNA, and 20.9 loci per subject with a mean of 6.7 spacers per locus from the DNA (Additional file 1: Table S6). The shortest loci for all subjects and CRISPR spacer types contained 2 spacers, and the longest SGI CRISPR locus contained 33 spacers, while the longest SGII CRISPR locus contained 60 spacers. For the majority of CRISPR loci produced, they were identical between the DNA and the cDNA, suggesting that in most cases the entire CRISPR locus was transcribed.

While there were relatively few spacers shared between most subjects (Fig. 2), a few subjects did share a significant proportion of their spacers (Additional file 2: Figure S14). For example, subjects H7 and H9 shared 45.1 % of their SGII spacers (Panel B), which corresponded to shared spacers amongst 9 separate assembled loci. There also were a significant number of SGII spacers (25.1 %) shared between subjects H7 and D4, which corresponded to shared spacers amongst 4 different loci. There also were significant proportions of GHI spacers shared between subjects H3 and H4, H3 and D4, H3 and D5, and H6 and D5 (Panel C); a high proportion (31.1 %) of VSI spacers were shared between subjects H1 and D6 (Panel D).

**Fig. 7:**
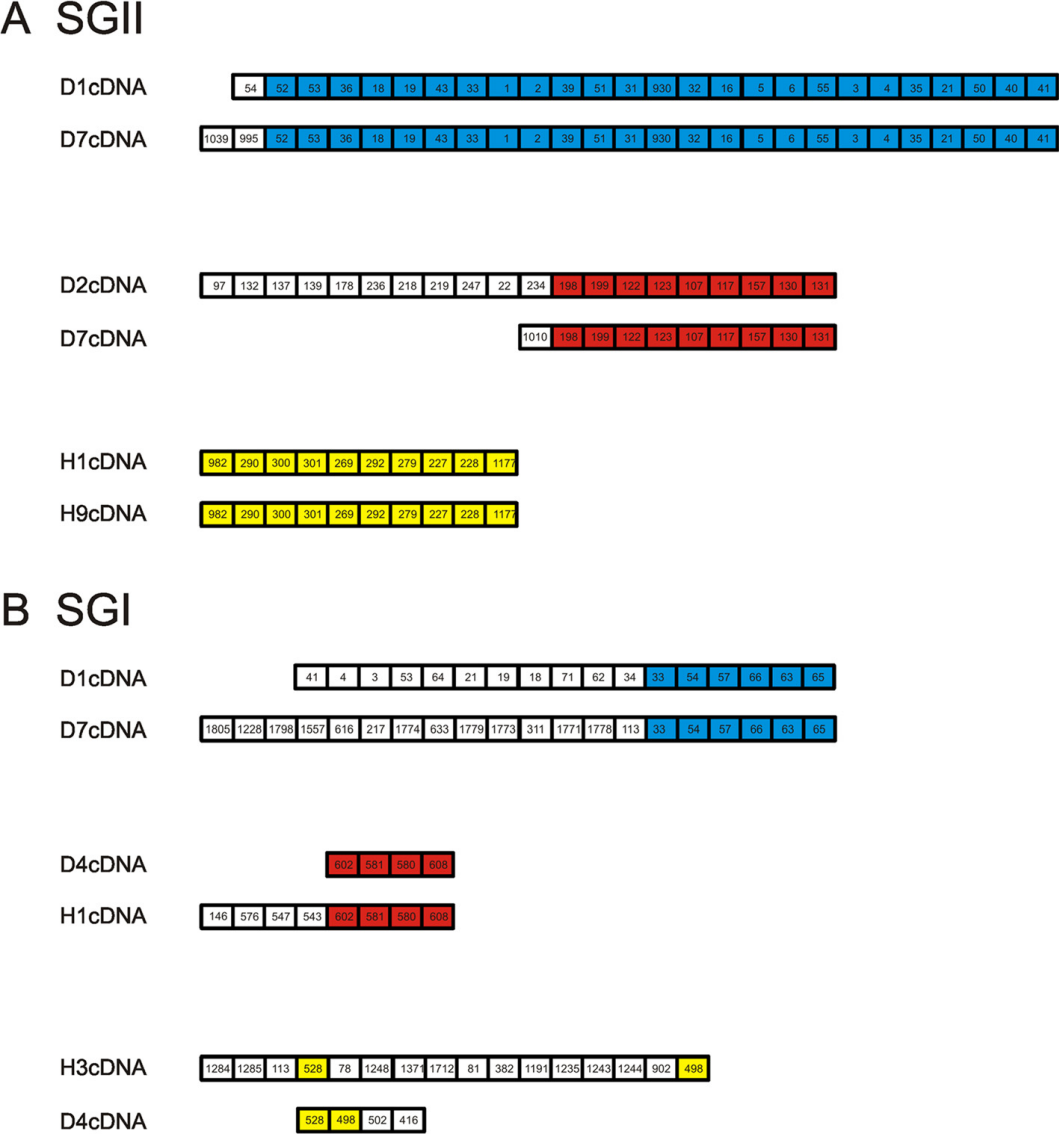
Reassembled CRISPR loci from various subjects. Panel **A** shows some representative loci from SGII loci and Panel **B** shows some representative loci from SGI loci. Each spacer is labeled with a unique numeric identifier for SGI and SGII. Spacers that are shared between different subjects are shown in colors, and white boxes represent spacers that are unique to that individual subject. The loci are drawn in a presumptive 5’ to 3’ orientation based on the end of the loci that differs between subjects

We evaluated the pattern of shared spacers between different CRISPR loci to decipher whether many of the spacers were shared as a result of separate phage exposures or whether they may have been inherited as a unit. We found several different patterns of SGI and SGII shared spacers amongst the reassembled CRISPR loci (Fig. 7), including: 1) loci that likely were inherited as a unit and continued to acquire new and different spacers, 2) loci that were identical and may no longer be acquiring new spacers, and 3) loci that have spacers with altered order that likely were acquired through separate exposures to similar phage.

### CRISPR loci matches to virome reads

**Fig. 8:**
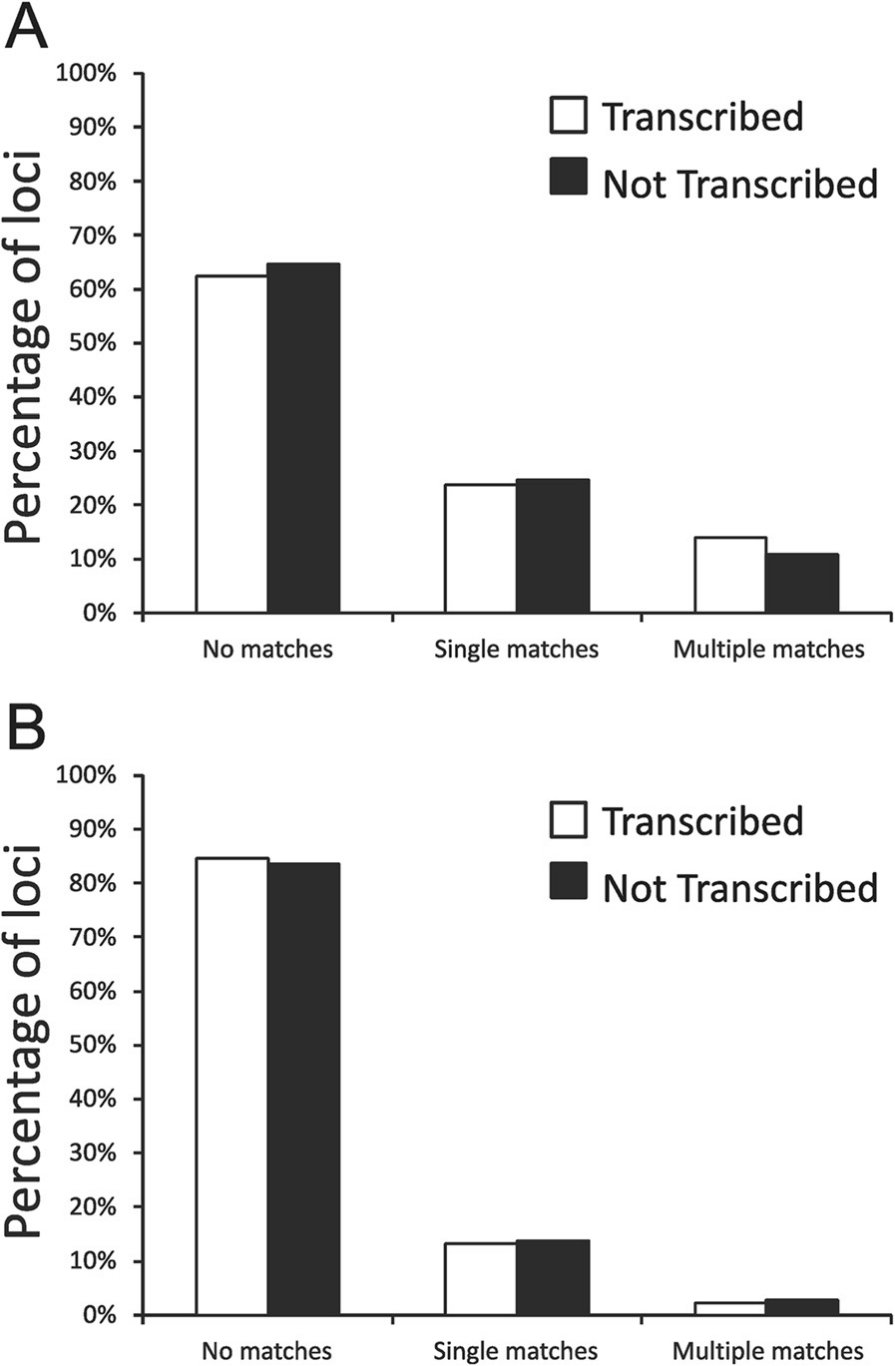
Percentage of reassembled CRISPR loci with spacers that match virome reads. The CRISPR loci are sorted based on those loci without matches, loci with only a single spacer that matches virome reads, and loci with multiple spacers that match virome reads. Transcribed loci are shown in white and those loci not identified in transcripts are shown in black. Panel **A** represents SGI spacers and Panel **B** represents SGII spacers

We quantified the number of CRISPR loci from all subjects that had spacers with matches in the viromes of the study subjects to determine whether there may be biases between those loci that matched phage based on whether they were transcribed. For SGII spacers, approximately 65 % of the reconstructed loci had no spacers that matched any virome reads, and there was no significant difference based on whether the locus was transcribed (Fig. 8, Panel A). Approximately 25 % only had single spacers that matched phage, while approximately 10 % had multiple spacers that matched phage. These numbers were significantly different for SGI spacers, where 85 % had no matches, 12 % had single matches, and 3 % had multiple spacers that matched viruses. Each subject had SGI and SGII CRISPR loci with matches to phage (Additional file 2: Figure S15), suggesting that in each subject there were CRISPR loci transcribed that may have been active in resistance against these phage. That there were no significant differences in the proportions of loci that matched phage based on whether or not the locus was transcribed, suggests that CRISPR-mediated resistance on a global scale is not regulated based on locus transcription.

We also tested whether the presence of spacers that matched viruses were concentrated to individual CRISPR loci, which might suggest that some individual loci may be more active against circulating viruses. We measured the proportion of CRISPR loci that only had single spacers that matched viruses and the proportion of CRISPR loci that had multiple matches to viruses. Interestingly, there was a significant (p < 0.05) proportion of SGII CRISPR loci that had a relatively high ratio of total spacers to virome matches (Additional file 2: Figure S16), suggesting that these particular loci were highly adapted to counteracting oral viruses. Similar results were not found for SGI spacers, however, the proportion of CRISPR spacers that matched viruses was much lower for SGI than SGII CRISPR spacers.

## Discussion

We previously examined CRISPR spacers from the oral cavities of human subjects using repeat-based amplification [11, 28, 29, 34, 35], which allowed us to examine large spacer repertoires from multiple different bacterial species simultaneously. In those studies, we could only characterize the presence of CRISPR spacers, but could not assemble them into loci nor could we determine whether or not those CRISPR loci were expressed. Here, we provide the first characterization of CRISPR spacers that are actively transcribed in the oral cavities of a cohort of human subjects, and demonstrate that the vast majority of the spacers present are actually transcribed. We amplified and characterized CRISPR spacers from 4 separate repeat motifs in different oral species of *Streptococcus*, *Gemella*, and *Veillonella*, and most of the spacers identified from each motif were transcribed (Fig. 3, Panel A). The transcription of most of these spacers is interesting in light of a recent study that showed tolerance to temperate phage via transcription-dependent targeting by the CRISPR-Cas system [54]. Most of the phage identified in the viromes in this study were predicted to have temperate lifestyles [33], which suggests that tolerance to temperate phage may exist even in the presence of basal CRISPR locus transcription. Some of these transcribed loci likely belong to the same bacteria, which supports that CRISPR-Cas systems may function independently in some bacteria [55].

The high proportion of spacers from each different repeat motif that were transcribed suggests that most of the CRISPR spacers in the oral cavity have some basal level of transcription. These results are in support of a prior study that showed the expression of CAS proteins from CRISPR-Cas systems that were previously thought to be inactive [56]. In that study, the expression of CAS proteins was increased in response to phage, and our finding of highly expressed CRISPR spacers suggests that the transcription of these loci may also be a response to the presence of phage. Transcription is a paramount early step in CRISPR-mediated resistance against plasmids and phage, but the transcription of CRISPR loci does not necessarily indicate CRISPR-mediated resistance. Depending on the organism and the CRISPR-Cas system type [57], other steps including the processing of transcripts into mature crRNAs [51–53], and presence/expression of other Cas genes [51, 58], ultimately determine whether the production of CRISPR locus transcripts are active in mediating resistance against viruses and plasmids. The presence of highly expressed spacers would suggest that there were phage present in the oral cavities of these subjects that stimulated transcription, yet our analysis did not demonstrate any relationship between highly expressed spacers and matches to oral phage. There are several possible explanations for this finding, including: 1) basal levels of transcription of these loci are sufficient to counteract invading phage and plasmids, 2) transcription of these loci may respond to other signals besides the presence of matching phage/plasmids and 3) lesser abundant phage that were not identified in our analysis may stimulate CRISPR locus transcription. We believe that the relative abundance may be very important in identifying phage that may stimulate CRISPR locus transcription in complex communities, as we found few assembled viruses in this analysis that matched spacers, but a far greater number of virome reads. This suggests that there were numerous viruses present in these viromes that could not be assembled due to a relatively low abundance or limitations in the assembly process. A longitudinal analysis of the CRISPRs and viromes may also highlight that some viruses in these communities fluctuate in their relative abundance, potentially as a result of interactions with CRISPRs.

The proportion of CRISPR spacers matching viruses in the oral cavity was relatively low, but differed significantly by CRISPR spacer type (Fig. 3, Panel B). Both SGI and SGII CRISPRs are found in multiple different oral streptococcal species [35] and represent Type II CRISPR-Cas systems [57], yet 3x the number of SGII spacers matched oral viruses than SGI spacers. The GHI spacers known to be found in oral species of *Gemella* and the VSI spacers found in oral species of *Veillonella* had many fewer spacers matching oral viruses when compared to streptococcal spacers. These data could reflect a high abundance of streptococcal phage present in each subject, relative abundances of different phage in the oral cavity, or potentially reflect the relative activity of these different CRISPR types in acquired resistance against oral phage. The data for individual spacers matching phage were also reflected in the relative proportions of reassembled CRISPR loci containing spacers that matched oral viruses. Approximately 35 % of the reassembled SGII CRISPR loci matched viruses, while only 15 % of SGI reassembled loci matched viruses (Fig. 8). Despite the increased proportion of SGII spacers matching the viruses, there was no bias identified in transcribed loci matching viruses. In fact, whether individual spacers or whole loci were identified in transcripts, had no effect upon the proportion of spacers or loci that matched viruses (Figs. 3 and 8). There also were some SGII loci that were extraordinarily well adapted to oral phage populations with all or nearly all their transcribed spacers matching oral viruses (Additional file 2: Figure S16). The highly individual-specific nature of the spacer repertoires in the subjects studied (Fig. 2) likely is related to bacterial strains present in each subject. We believe this explains why there was no association between spacer repertoires in periodontal health or disease (Additional file 1: Table S2), and there also were no specific patterns observed of spacers matching phage in these subjects (Additional file 2: Figures S4 and S5).

The CRISPR-Cas system for bacteria serves the important functions of providing acquired resistance against invading phage and plasmids. This study was designed to characterize those CRISPR spacers that matched phage in the oral cavities of the cohort of subjects; however, it could not address whether there might exist transcription biases against plasmids. Our prior studies have indicated that a small proportion of SGI and SGII spacers do match known streptococcal plasmids; however, we do not have metagenome data for plasmids present in the oral cavities of our subjects for comparison. We believe that the trends seen in spacers matching phage will be similar for plasmids; however, further studies are necessary to demonstrate that phenomenon.

There are several benefits of the repeat-based amplification approach utilized to characterize CRISPR locus expression in our study subjects. The first is that we can analyze multiple different CRISPR loci simultaneously without knowing their genomic locations in their various oral bacteria. Using this technique, we likely can analyze the CRISPR loci in tens to hundreds of different oral bacterial species rather than concentrating on a few individual species. Second, by utilizing longer sequencing reads, we can assemble CRISPR loci and improve our understanding of the various loci that target human oral viruses. While it is unlikely that we were able to assemble all CRISPR loci harboring the various repeat motifs, we were able to assemble many different loci with different spacer content. The patterns of shared spacers amongst the assembled loci suggests that some were inherited as a unit and continue to diversify, some were inherited and may no longer be diversifying, and that others are the result of separate phage exposures (Fig. 7). The mean number of CRISPR loci reassembled and the mean number of spacers per locus in each subject was highly similar for both SGI and SGII CRISPRs. Despite those similarities, the proportion of SGI and SGII spacers matching oral viruses was highly disparate. There were approximately 10 % more SGII than SGI spacers transcribed (Fig. 3, Panel B), but the proportion of transcribed spacers of neither CRISPR type was related to matching phage (Fig. 8). Further study is needed to evaluate whether the disparity between SGII and SGI may be related to their activities against oral phage.

## Conclusions

There are substantial repertoires of CRISPRs in the human oral cavity that likely are involved in acquired resistance against oral phage. Prior studies have shown that oral CRISPR repertoires have evolved specific adaptations to oral phage populations [28]. The human oral virome is populated by numerous different bacteriophage genotypes [25], and many of those phage likely are targeted by the various CRISPR types analyzed in this study. We had very little insight into whether CRISPR locus transcription at the community level is adapted to local virus populations, and the data presented in this study indicated that the vast majority of the oral CRISPR loci sampled were transcribed. The significant proportions of loci that were transcribed indicated that most were transcribed at a basal level, yet some spacers also were highly expressed. Loci that were highly transcribed were no more likely than others to have spacers that matched oral phage (Fig. 8), which could indicate that the phage to which they respond are of low relative abundance or that the transcription of these loci was responding to signals other than the presence of oral phage. There were no associations between oral health status and CRISPR spacer repertoires. The unique spacer repertoires found in all subjects suggest that the CRISPR loci in each subject have evolved as a result of their unique viral exposures. That many of the CRISPR loci in the human oral cavity were transcribed and match oral phage, suggests they may play a substantial role in shaping the human oral virome.

## Methods

### Ethics statement

All subjects were recruited and enrolled from the Western University College of Dental Medicine and their participation was approved by the Western University College of Dental Medicine and the University of California, San Diego Administrative Panels on Human Subjects in Medical Research. All subjects signed a written informed consent indicating their willingness to participate in this study.

### Human subjects

Each subject underwent a baseline periodontal examination that included measurements of probing depths, clinical attachment loss, Plaque Index, Gingival Index, and gingival irritation. We used the 1999 International Workshop for Classification of Periodontal Diseases and Conditions, where periodontal disease is defined by loss of attachment. For a diagnosis of healthy, all sites had to have an attachment level of 0 mm and an absence of bleeding on probing. Each subject completed a survey self-reporting his or her oral health, and any other pre-existing medical conditions that could result in substantial immunosuppression. Exclusion criteria included antibiotic administration during the month prior to the beginning of the study. Each subject provided a minimum of 3 ml of non-stimulated saliva, which was immediately frozen at −80 °C prior to use in this study.

### Amplification and binning of streptococcal CRISPR spacers

From each subject, genomic DNA was prepared from saliva using the Qiagen QIAamp DNA MINI Kit (Qiagen, Valencia, CA), with the addition of a bead beating step using Lysing Matrix B (MPBio, Solon, OH) prior to DNA extraction. CRISPR sequences were amplified based on primers designed from the palindromic repeat sequences of various CRISPRs (Additional file 1: Table S7). Primers pairs for SGI, SGII, GHI, and VSI CRISPR spacers contain 10-nucleotide barcode sequences (represented by the ‘X’), and were used to amplify CRISPR sequences from each subject (Additional file 1 Tables S7 and S8). Reaction conditions included 44 μl Platinum High-Fidelity PCR Mastermix (Invitrogen, Carlsbad, CA), 1 μl of each the forward and reverse primer (10 mmol each), and 4 μl DNA template. The following were used as cycling parameters: 2 min initial denaturation at 94 °C, followed by 30 cycles of denaturation (15 s at 95 °C), annealing (15 s), and extension (2 min at 72 °C), followed by a final extension (10 min at 72 °C). CRISPR amplicons were gel extracted using the Qiagen MinElute Kit (Qiagen, Valencia, CA). Molar equivalents were determined for each product using an Agilent Bioanalyzer HS DNA Kit (Agilent, Santa Clara, CA), and each were pooled into equimolar equivalents. Resulting pools were sequenced on 314 chips using an Ion Torrent PGM according to manufacturer’s instructions (Life Technologies, Grand Island, NY) [59]. Barcoded sequences then were binned according to 100 % matching barcodes. Each read was trimmed according to modified Phred scores of 0.5, and low complexity reads and reads with ambiguous characters were removed prior to further analysis. Only those reads that had 100 % matching sequences to both the 5’ and the 3’ end of the CRISPR repeat motifs were used for further evaluation. Spacers were defined as any nucleotide sequences (length ≥20) in between repeat motifs. To group spacers according to their trinucleotide content, we first compiled the trinucleotide content for all spacers and added them to a database. For each sequence, the difference in trinucleotide content was compared between all possible pairs of sequences regardless of overall spacer length, as length differences between identical spacers over the length of the shorter spacer would only account for small differences in total trinucleotide content. The sum of the differences for all sequence pairs then was determined, and then spacers were binned together if their differences were less than the standard deviation from the mean overall difference. Charts were created that included the total number of spacers with a specified number of trinucleotide differences (Additional file 2: Figure S1). To validate the technique, random datasets of spacers were created with known numbers of mutations (including indels and polymorphisms). This technique was validated as previously described [29].

For each subject evaluated, a database of spacer groups was generated, and databases were compared to determine shared spacer groups and to create heatmaps using Java Treeview [60]. CRISPR spacers for each subject were used to search a database of the virome reads for matches from all viromes combined, and the number of spacer matches per virome read was used to create heatmaps. Spacers from each subject were subjected to BLASTN analysis based on the NCBI non-redundant database. Hits were considered significant based on bit scores ≥45, which roughly correlated to 2 nucleotide differences over the length of a 30 nucleotide spacer. CRISPR spacers for each subject were used to search a database of the virome reads for matches from all viromes combined. Matches to viromes were defined as exact matches to any spacer sequence within any spacer group. Rarefaction analysis was performed based on spacer group richness estimates of 10,000 iterations using EcoSim [61].

### Identification of overexpressed spacers

To identify those CRISPR spacers with high expression, we compared the CRISPR spacer repertoires found in the DNA fractions with those found in the cDNA. The relative abundances of spacers in both fractions were normalized according to their Percentage Per Thousand Spacers (PPTS) so that the relative proportions of spacers could be compared directly between DNA and cDNA. Spacers were ranked according to their relative abundances in the DNA and cDNA fractions and the ranks used as input for determinations of Spearman’s rho according to the formula 1-((6-∑d_i_^2^)/n(n^3^-1)), where d_i_ represents the difference between the ranks and n is the total number of spacers compared. We utilized the PPTS values as baseline expression values and determined residuals by subtracting the PPTS for cDNA from baseline. Each residual was then compared back to the baseline value and those spacers in which the residuals represented 3x the baseline DNA value was considered highly expressed. Only those spacers with baseline values of ≥5 were considered in the analysis to determine highly expressed spacers. The distribution of all the PPTS values for both the DNA and cDNA are shown in Additional file 2 Figures S6, S7, S8 and S9.

### CRISPR locus assembly

To assemble CRISPR loci, we binned all spacer sequences according to their trinucleotide content. We then assigned a unique identifier to each spacer group identified and assigned the spacers within each read with that unique identifier. We then compiled a database of all reads by their unique identifiers and determined the frequency that each combination of spacers were adjacent to one another based on the proportion of times they could be adjacent given their known frequencies within the dataset. For spacer combinations that had adjacent spacers in which their frequency was greater than 60 %, they were assembled iteratively by matching adjacent spacers in the reads. For most loci, the relative frequencies of adjacent spacers within the locus were within 15 % for all spacer combinations. For those spacer combinations in which their frequency was <60 % but >20 %, they were assembled manually, as in most cases they could be placed into alternative loci that likely represented CRISPR loci of lower relative abundance. For each subject, the loci assembled manually were verified by designing primers for the first and last spacer in each locus followed by PCR amplification. A few of the PCR amplicons also were subjected to Sanger sequencing to ensure that their actual sequence matched that which was reassembled from the short read data (Additional file 2 Figures S11 and S12).

### Statistical analysis

To assess whether spacer groups had significant overlap between the DNA and cDNA of each subject, we performed a permutation test. We simulated the distribution of the fraction of shared spacers between different individuals and nucleic acid types. For each set, we computed the summed fraction of randomly chosen spacer groups across different subjects and nucleic acid types, and from those computed an empirical null distribution of statistics. The fraction computed resulted from 10,000 iterations, and included 1000 spacer groups sampled per iteration. The standard deviation was computed from the percentage of shared spacer groups over the 10,000 iterations. For each subject, an empirical null distribution of statistics was determined. The observed statistic was referred to this distribution, and the *p* value was computed as the fraction of times the simulated statistic for intra-subject exceeded the simulated statistic for the inter-subject.

Comparisons of the mean percentages of shared spacers and standard error rates in different subjects or between groups of subjects were performed using Microsoft Excel 2007 (Microsoft Corp., Redman, WA). Statistical significance was determined by two-tail t-test for comparison of means.

## Additional files

Additional file 1: Table S1. Numbers of CRISPR-bearing reads and spacers sequenced per subject. **Table S2.** Estimates of shared CRISPR spacers by oral health status or nucleic acid type. **Table S3.** Estimates of shared CRISPR spacers within and between subjects. **Table S4.** Numbers of CRISPR-bearing longer reads and spacers sequenced per subject. **Table S5.** Reassembled SGI CRISPR loci from all subjects. **Table S6.** Reassembled SGII CRISPR loci from all subjects. Additional file 2: Figure S1. Plots of CRISPR spacer comparisons for each CRISPR spacer type. The Y-axis represents the number of CRISPR spacer comparisons performed and the X-axis represents the number of trinucleotide differences. The dashed line corresponds to the statistical cutoff for binning CRISPR spacers into spacer groups. Panel A—SGI CRISPR spacers, Panel B—SGII CRISPR spacers, Panel C—GHI CRISPR spacers, and Panel D—VSI CRISPR spacers. **Figure S2.** Rarefaction analysis of CRISPR spacer groups in the saliva of all subjects. Rarefaction curves were created using 10,000 random iterations based on spacer group richness. The y-axis represents the number of unique spacer groups and the x-axis represents the number of spacers sampled. Panel A—SGI CRISPRs, Panel B—SGII CRISPRs, Panel C—GHI CRISPRs, and Panel D—VSI CRISPRs. Periodontally healthy subjects are shown in white and subjects with significant periodontal disease are shown in black. Triangles represent CRISPR spacers from cDNA and circles represent CRISPR spacers from genomic DNA. **Figure S3.** Estimated percentages (± standard deviation) of CRISPR spacers shared between genomic DNA and cDNA. Within subject comparisons are demonstrated by black bars and between subject comparisons are demonstrated by the white bars. Panel A—SGI CRISPR spacers, Panel B—SGII CRISPR spacers, Panel C—GHI CRISPR spacers, and Panel D—VSI CRISPR spacers. **Figure S4.** Heatmaps of SGI (Panel A) and SGII (Panel B) CRISPR spacers that match virome reads. Virome reads from subjects with relative periodontal health and those with periodontal disease are shown along the rows. The columns represent the different subjects with periodontal health or disease. **Figure S5.** Principal coordinates analysis of beta diversity between subjects with CRISPR spacer matches to virome reads. Subjects with relative periodontal health are shown by white circles and subjects with periodontal disease are shown by black circles. Panel A—SGI CRISPRs, Panel B—SGII CRISPRs, Panel C—GHI CRISPRs, and Panel D—VSI CRISPRs. **Figure S6.** Plots of PPTS (Percentage Per Thousand Spacers) values for SGI spacers from the DNA fractions (black diamonds) and the cDNA fractions (red boxes). The PPTS values are shown on the y-axis and the individual spacers are shown on the x-axis. Each box or diamond represents a different individual spacer. Panel A—H represent subjects with relative periodontal health and Panels I-O represent subjects with periodontal disease. **Figure S7.** Plots of PPTS (Percentage Per Thousand Spacers) values for SGII spacers from the DNA fractions (black diamonds) and the cDNA fractions (red boxes). The PPTS values are shown on the y-axis and the individual spacers are shown on the x-axis. Each box or diamond represents a different individual spacer. Panel A-H represent subjects with relative periodontal health and Panels I-O represent subjects with periodontal disease. **Figure S8.** Plots of PPTS (Percentage Per Thousand Spacers) values for GHI spacers from the DNA fractions (black diamonds) and the cDNA fractions (red boxes). The PPTS values are shown on the y-axis and the individual spacers are shown on the x-axis. Each box or diamond represents a different individual spacer. Panel A-H represent subjects with relative periodontal health and Panels I-O represent subjects with periodontal disease. **Figure S9.** Plots of PPTS (Percentage Per Thousand Spacers) values for VSI spacers from the DNA fractions (black diamonds) and the cDNA fractions (red boxes). The PPTS values are shown on the y-axis and the individual spacers are shown on the x-axis. Each box or diamond represents a different individual spacer. Panel A-H represent subjects with relative periodontal health and Panels I-O represent subjects with periodontal disease. **Figure S10.** Bar plots of the percentages (± standard deviation) of CRISPR spacers that are highly expressed. The x-axis represents the CRISPR spacer type and the y-axis represents the percentage of all spacers. **Figure S11.** Sequence of reassembled CRISPR locus from healthy subject H5. **Figure S12.** Sequence of reassembled CRISPR locus from subject D1 with periodontal disease. **Figure S13.** Rarefaction analysis of CRISPR spacer groups from 200bp reads in the saliva of all subjects. **Figure S14.** CRISPR spacer group heat matrices demonstrating the percentage of shared CRISPR spacer groups between all subjects. The top triangular portion of each matrix represents comparisons between CRISPR spacers derived from subjects with relative periodontal health, the bottom rectangular portion of each matrix represents comparisons between periodontal health-derived and periodontal disease-derived CRISPR spacers, and the bottom triangular portion of each matrix represents comparisons between periodontal disease-derived CRISPR spacers. The intensity scale bar is located to the right. Panel A—SGI, Panel B—SGII, Panel C—GHI, and Panel D—VSI CRISPR spacers. **Figure S15.** Percentage of reassembled CRISPR loci with spacers that match virome reads for subjects with periodontal health and disease. **Figure S16.** Ratio of CRISPR spacer matches to total spacer counts in reassembled loci. The ratio of matches to spacer counts is shown for loci with multiple spacers that match viromes (white boxes) and for those with only single spacers that match virome reads (black boxes). The mean ratio for all loci from all subjects is indicated by the black bar. SGII CRISPR loci are located on the left, and SGI loci are located on the right.

## Abbreviations

CRISPR: Clustered regularly interspaced short palindromic repeats
PPTS: Percentage per thousand spacers
NR: Non-redundant
crRNAs: CRISPR RNAs
CAS: CRISPR associated
SGI: Streptococcus Group I
SGII: Streptococcus Group II
GHI: Gemella haemolysans Group I
VSI: Veillonella species Group I
