# Supplementary material for: Global transcription of CRISPR loci in the human oral cavity
Source: BMC Genomics. 2015 May 21;16(1):401. doi: 10.1186/s12864-015-1615-0 (PMC4438527; doi:10.1186/s12864-015-1615-0)
Supplement: Additional file 1: Table S1. — Numbers of CRISPR-bearing reads and spacers sequenced per subject. Table S2. Estimates of shared CRISPR spacers by oral health status or nucleic acid type. Table S3. Estimates of shared CRISPR spacers within and between subjects. Table S4. Numbers of CRISPR-bearing longer reads and spacers sequenced per subject. Table S5. Reassembled SGI CRISPR loci from all subjects. Table S6. Reassembled SGII CRISPR loci from all subjects. [file 12864_2015_1615_MOESM1_ESM.pdf]

Supplemental Table 1a: SGI CRISPR Spacer Sequences

	DNA				cDNA			
**Subject**	**Reads**	**Spacers**	**Spacer Groups**	**Mean Length**	**Reads**	**Spacers**	**Spacer Groups**	**Mean Length**
H1	23446	17118	277	30	13514	9098	228	30
H2	11441	8549	132	30	16248	12159	134	30
H3	26559	14441	106	30	17490	5734	81	30
H4	37199	8950	174	30	14593	10213	169	30
H5	32906	9270	203	30	15780	10191	203	30
H6	46906	14837	318	30	28922	21607	396	30
H7	45107	36644	189	30	29783	18677	344	30
H8	19286	14462	331	30	21629	15401	424	30
H9	20413	14890	311	30	21329	7188	213	30
D1	12146	4363	91	30	59854	44802	156	30
D2	29399	13047	219	30	22885	5815	170	30
D3	13245	2294	47	30	16494	4959	59	30
D4	41923	24209	278	30	16338	7665	153	30
D5	22200	19523	210	30	19406	16089	221	30
D6	12230	10091	236	30	19959	14458	279	30
D7	14469	12208	419	30	18655	15057	464	30

Supplemental Table 1b: SGII CRISPR Spacer Sequences

	DNA				cDNA			
**Subject**	**Reads**	**Spacers**	**Spacer Groups**	**Mean Length**	**Reads**	**Spacers**	**Spacer Groups**	**Mean Length**
H1	19659	13047	198	30	58934	36315	236	30
H2	18732	11875	246	30	12137	5368	139	30
H3	15041	12484	127	30	12939	9386	133	30
H4	19867	13990	151	30	15554	10151	151	30
H5	27595	13078	299	30	15300	10515	286	30
H6	46906	16365	291	30	14369	9351	238	30
H7	11434	8235	169	30	11618	6685	169	30
H8	22619	16007	273	30	12380	9525	250	30
H9	26187	19733	215	30	14942	11219	183	30
D1	35007	25542	68	30	19235	10368	62	30
D2	72693	50743	259	30	28225	18793	238	30
D3	30085	21681	67	30	26158	19185	77	30
D4	37866	27552	148	30	10092	7404	129	30
D5	11051	7416	200	30	14211	9380	206	30
D6	13013	9429	147	30	14255	9752	155	30
D7	37199	15726	301	30	20058	11919	320	30

Supplemental Table 1c: GHI CRISPR Spacer Sequences

	DNA				cDNA			
Subject	Reads	Spacers	Spacer Groups	Mean Length	Reads	Spacers	Spacer Groups	Mean Length
H1	24113	22539	165	30	17954	17061	137	30
H2	26611	24393	267	30	17009	15299	166	30
H3	21199	18678	77	30	11969	11212	68	30
H4	21496	19388	80	30	13617	12569	67	30
H5	40690	37721	115	30	28348	26742	85	30
H6	24161	21718	275	30	66647	63776	389	30
H7	13084	12319	159	30	19905	18235	116	30
H8	10569	8892	138	30	14168	11950	140	30
H9	18886	16418	128	30	44552	41488	159	30
D1	36185	34529	111	30	22052	20466	58	30
D2	25092	23192	209	30	25854	24472	147	30
D3	16257	15177	42	30	38570	36381	48	30
D4	26149	24418	64	30	12908	12107	85	30
D5	40214	36349	154	30	21489	20512	313	30
D6	19176	18235	100	30	11019	10301	99	30
D7	70263	63721	331	30	10694	9500	224	30

Supplemental Table 1d: VSI CRISPR Spacer Sequences

	DNA				cDNA			
**Subject**	**Reads**	**Spacers**	**Spacer Groups**	**Mean Length**	**Reads**	**Spacers**	**Spacer Groups**	**Mean Length**
H1	12281	9628	95	37	60930	55804	150	37
H2	26097	18856	192	37	18216	13592	190	37
H3	33459	26740	46	37	11655	8440	24	37
H4	64865	46454	41	37	15096	10619	49	37
H5	18703	14903	127	37	15689	12049	136	37
H6	57965	51071	169	37	13641	12383	131	37
H7	85020	61345	359	37	14435	10747	191	37
H8	12004	9117	148	37	77485	61222	333	38
H9	17138	12967	266	37	16879	11544	181	37
D1	20397	14993	52	37	32499	30565	69	37
D2	48114	40667	167	36	15340	13154	113	36
D3	48202	41272	79	38	31008	26318	70	37
D4	33650	25848	92	36	13163	8823	81	37
D5	31198	24645	78	38	10911	8373	56	38
D6	29986	23069	72	37	12076	10728	31	37
D7	52927	41793	107	37	12142	9403	104	37

Supplemental Table 2. Shared CRISPR spacers by health or nucleic acid status

By Health Status	Percent Homologous within each group[a]	Percent Homologous between each group[a]	P-value[b]
SGI	13.52 ± 6.90	11.11 ± 6.26	0.3824
SGII	12.77 ± 4.94	10.89 ± 5.15	0.3786
GHI	21.12 ± 5.91	21.36 ± 6.74	0.5035
VSI	12.45 ± 7.90	15.01 ± 8.80	0.5895
**By nucleic acid**			
SGI	8.90 ± 7.25	8.33 ± 5.81	0.5056
SGII	9.88 ± 5.10	9.39 ± 5.36	0.4579
GHI	15.39 ± 6.47	14.48 ± 6.24	0.4562
VSI	10.35 ± 8.51	9.57 ± 9.20	0.4615

[a]Based on the mean of 10,000 iterations. 1,000 random spacers were sampled per iteration

[b]Empirical p-value based on the fraction of times the estimated percent shared spacers with each group exceeded that between groups

Supplemental Table 3a. SGI CRISPR spacers shared within and between subjects

By Subject	Percent Homologous Intra-subject[a]	Percent Homologous Inter-subject[a]	P value[b]
D1	81.97 ± 1.90	18.75 ± 5.10	**<0.0001**
D2	88.84 ± 1.30	12.80 ± 2.42	**<0.0001**
D3	88.39 ± 1.12	24.38 ± 5.53	**<0.0001**
D4	59.89 ± 3.24	19.47 ± 3.91	**<0.0001**
D5	98.83 ± 0.12	9.62 ± 2.12	**<0.0001**
D6	95.27 ± 0.45	13.61 ± 3.70	**<0.0001**
D7	96.81 ± 0.33	18.15 ± 4.58	**<0.0001**
H1	98.63 ± 0.13	15.18 ± 2.84	**<0.0001**
H2	98.47 ± 0.14	15.54 ± 2.25	**<0.0001**
H3	94.44 ± 0.58	12.40 ± 2.32	**<0.0001**
H4	99.37 ± 0.07	15.19 ± 2.89	**<0.0001**
H5	94.76 ± 0.53	17.30 ± 3.63	**<0.0001**
H6	97.54 ± 0.24	14.37 ± 3.05	**<0.0001**
H7	85.64 ± 2.05	16.12 ± 4.65	**<0.0001**
H8	96.58 ± 0.31	16.51 ± 3.91	**<0.0001**
H9	96.49 ± 0.43	20.99 ± 3.83	**<0.0001**

[a]Based on the mean of 10,000 iterations. 1,000 random spacers were sampled per iteration

[b]Empirical p-value based on the fraction of times the number of shared spacers within each subject exceeds that for different subjects. Bold represents p-values <0.05.

Supplemental Table 3b. SGII CRISPR spacers shared within and between subjects

By Subject	Percent Homologous Intra-subject[a]	Percent Homologous Inter-subject[a]	P value[b]
D1	99.41 ± 0.05	15.74 ± 1.85	**<0.0001**
D2	99.24 ± 0.07	12.44 ± 1.95	**<0.0001**
D3	99.13 ± 0.12	9.82 ± 2.11	**<0.0001**
D4	98.63 ± 0.15	10.33 ± 2.01	**<0.0001**
D5	98.00 ± 0.17	22.48 ± 3.43	**<0.0001**
D6	98.77 ± 0.12	22.63 ± 4.21	**0.0127**
D7	97.57 ± 0.28	19.86 ± 3.16	**<0.0001**
H1	98.72 ± 0.47	15.07 ± 2.01	**<0.0001**
H2	89.72 ± 0.93	20.41 ± 3.02	**<0.0001**
H3	99.71 ± 0.04	14.82 ± 2.56	**<0.0001**
H4	99.18 ± 0.08	21.56 ± 2.84	**<0.0001**
H5	98.40 ± 0.14	24.43 ± 3.51	**<0.0001**
H6	98.14 ± 0.17	19.44 ± 3.37	**<0.0001**
H7	97.58 ± 0.21	27.07 ± 3.38	**<0.0001**
H8	98.34 ± 0.16	17.16 ± 2.89	**<0.0001**
H9	98.59 ± 0.13	16.00 ± 2.76	**<0.0001**

[a]Based on the mean of 10,000 iterations. 1,000 random spacers were sampled per iteration

[b]Empirical p-value based on the fraction of times the number of shared spacers within each subject exceeds that for different subjects. Bold represents p-values <0.05.

Supplemental Table 3c. GHI CRISPR spacers shared within and between subjects

By Subject	Percent Homologous Intra-subject[a]	Percent Homologous Inter-subject[a]	P value[b]
D1	99.41 ± 0.07	14.83 ± 2.79	**<0.0001**
D2	99.12 ± 0.09	27.06 ± 3.69	**<0.0001**
D3	99.71 ± 0.02	15.73 ± 2.32	**<0.0001**
D4	99.58 ± 0.06	29.23 ± 4.29	**<0.0001**
D5	95.94 ± 0.45	25.38 ± 4.12	**<0.0001**
D6	99.68 ± 0.04	22.47 ± 3.33	**<0.0001**
D7	97.97 ± 0.23	18.72 ± 3.41	**<0.0001**
H1	98.34 ± 0.25	32.09 ± 3.52	**<0.0001**
H2	83.05 ± 1.50	30.44 ± 3.56	**<0.0001**
H3	99.71 ± 0.03	39.50 ± 2.78	**<0.0001**
H4	99.64 ± 0.04	35.69 ± 2.81	**<0.0001**
H5	96.03 ± 0.53	15.84 ± 2.55	**<0.0001**
H6	97.02 ± 0.31	25.52 ± 4.40	**<0.0001**
H7	95.29 ± 0.52	21.94 ± 4.36	**<0.0001**
H8	98.66 ± 0.19	27.89 ± 4.03	**<0.0001**
H9	98.44 ± 0.19	24.37 ± 3.59	**<0.0001**

[a]Based on the mean of 10,000 iterations. 1,000 random spacers were sampled per iteration

[b]Empirical p-value based on the fraction of times the number of shared spacers within each subject exceeds that for different subjects. Bold represents p-values <0.05.

Supplemental Table 3d. VSI CRISPR spacers shared within and between subjects

By Subject	Percent Homologous Intra-subject[a]	Percent Homologous Inter-subject[a]	P value[b]
D1	7.51 ± 2.73	16.86 ± 4.35	0.9714
D2	98.74 ± 0.17	17.49 ± 4.94	**<0.0001**
D3	99.48 ± 0.06	16.61 ± 3.14	**<0.0001**
D4	98.94 ± 0.14	20.75 ± 5.58	**<0.0001**
D5	96.54 ± 0.38	13.90 ± 3.91	**<0.0001**
D6	85.48 ± 1.32	26.87 ± 3.53	**<0.0001**
D7	98.80 ± 0.11	9.49 ± 3.70	**<0.0001**
H1	59.62 ± 4.13	16.83 ± 6.25	**<0.0001**
H2	97.47 ± 0.34	22.38 ± 5.31	**<0.0001**
H3	99.40 ± 0.10	19.21 ± 4.42	**<0.0001**
H4	99.43 ± 0.08	11.99 ± 2.25	**<0.0001**
H5	96.69 ± 0.34	24.02 ± 6.53	**<0.0001**
H6	99.05 ± 0.14	17.20 ± 5.52	**<0.0001**
H7	78.68 ± 2.29	9.30 ± 2.70	**<0.0001**
H8	86.57 ± 1.95	15.04 ± 7.10	**<0.0001**
H9	96.11 ± 0.46	32.65 ± 7.82	**<0.0001**

[a]Based on the mean of 10,000 iterations. 1,000 random spacers were sampled per iteration

[b]Empirical p-value based on the fraction of times the number of shared spacers within each subject exceeds that for different subjects. Bold represents p-values <0.05.

Supplemental Table 4a: SGI CRISPR Spacer Sequences

	DNA				cDNA			
**Subject**	**Reads**	**Spacers**	**Spacer Groups**	**Mean Length**	**Reads**	**Spacers**	**Spacer Groups**	**Mean Length**
H1	8911	16338	242	30	5514	10112	215	30
H2	8633	16638	142	30	11993	23421	117	30
H3	7056	13476	71	30	5809	11324	93	30
H4	9594	18135	156	30	20433	39091	169	30
H5	9139	17587	159	30	20914	40158	158	30
H6	10023	19434	312	30	17242	33207	335	30
H7	6746	12316	132	30	20236	37121	83	30
H8	11507	21964	324	30	9018	17451	376	30
H9	13631	25280	193	30	15119	28586	213	30
D1	7814	15074	129	30	5789	11218	129	30
D2	6456	11765	186	30	7147	13269	236	30
D3	7583	13943	67	30	21960	42667	77	30
D4	7320	13750	171	30	12510	24299	177	30
D5	12186	23581	216	30	8706	17056	210	30
D6	6578	12476	198	30	6335	12174	216	30
D7	6868	12992	347	30	7539	14471	396	30

Supplemental Table 4b: SGII CRISPR Spacer Sequences

	DNA				cDNA			
**Subject**	**Reads**	**Spacers**	**Spacer Groups**	**Mean Length**	**Reads**	**Spacers**	**Spacer Groups**	**Mean Length**
H1	9974	19400	207	30	8352	16390	197	30
H2	5608	10968	259	30	7028	13682	237	30
H3	5342	10820	87	30	6967	13864	88	30
H4	13136	25940	138	30	10666	20881	171	30
H5	13603	26453	361	30	11071	21372	356	30
H6	13838	27060	320	30	11400	22261	276	30
H7	13735	27011	240	30	21548	42506	251	30
H8	6394	12794	230	30	10827	21397	241	30
H9	8144	16102	222	30	6884	13493	190	30
D1	9122	17506	68	30	9334	17606	66	30
D2	7138	13946	230	30	9478	18466	233	30
D3	7417	14557	61	30	9852	19424	65	30
D4	5573	10899	135	30	7310	14236	133	30
D5	12447	24678	220	30	15198	29877	221	30
D6	12738	25240	164	30	10757	21229	156	30
D7	14003	27579	326	30	10712	21064	338	30

Supplemental Table 5: SGI CRISPR Loci

Subject	Nucleic Acid Type	Spacer Count	Spacer Groups	Number of Loci	Spacers Per Locus	Range
Periodontal Disease						
D1						
	cDNA	11218	129	16	5.5	2 to 17
	DNA	15074	129	17	5.2	2 to 17
D2						
	cDNA	13269	236	24	5.2	2 to 17
	DNA	11765	186	19	5.8	2 to 18
D3						
	cDNA	42667	77	2	10.0	2 to 18
	DNA	13943	67	4	14.0	2 to 33
D4						
	cDNA	24299	177	20	6.6	2 to 18
	DNA	13750	171	18	7.2	2 to 26
D5						
	cDNA	17056	210	27	5.1	2 to 15
	DNA	23581	216	19	5.7	2 to 18
D6						
	cDNA	12174	216	25	6.3	2 to 28
	DNA	12476	198	17	5.7	2 to 28
D7						
	cDNA	14471	396	45	4.5	2 to 20
	DNA	12992	347	40	4.4	2 to 15
Periodontal Health						
H1						
	cDNA	10112	215	38	3.5	2 to 22
	DNA	16338	242	34	3.9	2 to 22
H2						
	cDNA	23421	117	9	8.4	2 to 29
	DNA	16638	142	11	7.7	2 to 32
H3						
	cDNA	11324	93	14	4.8	2 to 16
	DNA	13476	71	9	5.2	2 to 10
H4						
	cDNA	39091	169	13	6.2	2 to 21
	DNA	18135	156	13	4.4	2 to 25
H5						
	cDNA	40158	158	18	5.9	2 to 22
	DNA	17587	159	16	4.9	2 to 20
H6						
	cDNA	33207	335	41	5.2	2 to 23
	DNA	19434	312	40	5.2	2 to 25
H7						
	cDNA	37121	83	12	4.6	2 to 18
	DNA	12316	132	19	3.6	2 to 17
H8						
	cDNA	17451	376	45	6.0	2 to 23
	DNA	21964	324	39	6.1	2 to 25
H9						
	cDNA	28586	213	28	4.3	2 to 14
	DNA	25280	193	21	5.0	2 to 13

Supplemental Table 6: SGII CRISPR Loci

Subject	Nucleic Acid Type	Spacer Count	Spacer Groups	Number of Loci	Spacers Per Locus	Range
Periodontal Disease						
D1						
	cDNA	17606	66	5	8.0	2 to 31
	DNA	17506	68	4	10.0	2 to 33
D2						
	cDNA	18466	233	11	13.0	2 to 27
	DNA	13946	230	14	9.5	2 to 28
D3						
	cDNA	19424	65	10	2.6	2 to 13
	DNA	14557	61	5	4.2	2 to 19
D4						
	cDNA	14236	133	20	5.5	2 to 33
	DNA	10899	135	20	4.8	2 to 33
D5						
	cDNA	29877	221	17	11.4	2 to 38
	DNA	24678	220	18	10.5	2 to 38
D6						
	cDNA	21229	156	9	9.2	2 to 19
	DNA	25240	164	12	8.1	2 to 17
D7						
	cDNA	21064	338	34	5.7	2 to 35
	DNA	27579	326	33	6.1	2 to 28
Periodontal Health						
H1						
	cDNA	16390	197	21	8.4	2 to 32
	DNA	19400	207	18	8.0	2 to 32
H2						
	cDNA	13682	237	34	5.7	2 to 17
	DNA	10968	259	42	4.4	2 to 15
H3						
	cDNA	13864	88	6	12.3	2 to 21
	DNA	10820	87	6	11.8	2 to 22
H4						
	cDNA	20881	171	21	5.9	2 to 17
	DNA	25940	138	15	4.7	2 to 20
H5						
	cDNA	21372	356	26	4.7	2 to 42
	DNA	26453	361	25	4.6	2 to 38
H6						
	cDNA	22261	276	24	6.3	2 to 32
	DNA	27060	320	35	5.2	2 to 36
H7						
	cDNA	42506	251	34	5.0	2 to 43
	DNA	27011	240	31	4.8	2 to 60
H8						
	cDNA	21397	241	31	6.4	2 to 23
	DNA	12794	230	29	5.1	2 to 22
H9						
	cDNA	13493	190	21	6.6	2 to 24
	DNA	16102	222	27	4.5	2 to 30

Supplemental Table 7. CRISPR Repeat Motifs and Primers

**Name**		**Consensus sequence and primer sequences**
**Streptococcus (SGI)**[a]	Consensus	GTTTTGGAACCATTCGAAACAACACAGCTCTAAAAC
	Forward	CCATCTCATCCCTGCGTGTCTCCGACTCAGXXXXXXXXXXCGTTTACGGTATTGAAAC[c]
	Reverse	CCTCTCTATGGGCAGTCGGTGATGGTAAGCACTGTTGCAAC
**Streptococcus (SGII)**[b]	Consensus	GTTGTACAGTTACTTAAATCTTGAGAGTACAAAAAC
	Forward	CCATCTCATCCCTGCGTGTCTCCGACTCAGXXXXXXXXXXCAGTTACTTAAATCTTGAGAG[c]
	Reverse	CCTCTCTATGGGCAGTCGGTGATAGATTTAAGTAACTGTACAAC
**Gemella haemolysans (GHI)**	Consensus	GTTTGAGAGATATGTAAATTTTGAATTCTACAAAAC
	Forward	CCATCTCATCCCTGCGTGTCTCCGACTCAGXXXXXXXXXXTTTTGAATTCTACAAAAC[c]
	Reverse	CCTCTCTATGGGCAGTCGGTGATTTTACATATCTCTCAAAC
**Veillonella sp3. (VSI)**	Consensus	GTTGCAACAGTGCTTACCGTTTACGGTATTGAAAC
	Forward	CCATCTCATCCCTGCGTGTCTCCGACTCAGXXXXXXXXXXCAGTTACTTAAATCTTGAGAG[c]
	Reverse	CCTCTCTATGGGCAGTCGGTGATAGTTTAAGTAACTGTACAAC

[a]Identified from Streptococcus mutans

[b]Identified from Streptococcus gordonii

[c]X's represent the location of the barcode sequence

Supplemental Table 8.

Barcode Adaptors Used for CRISPR Amplification

Barcode Number	Barcode Sequence
1	ACGAGTGCGT
2	ACGCTCGACA
3	AGACGCACTC
4	AGCACTGTAG
5	ATCAGACACG
6	ATATCGCGAG
7	CGTGTCTCTA
8	CTCGCGTGTC
9	TAGTATCAGC
10	TCTCTATGCG
11	TGATACGTCT
12	TACTGAGCTA
13	CATAGTAGTG
14	CGAGAGATAC
15	ATACGACGTA
16	TCACGTACTA
17	CGTCTAGTAC
18	TCTACGTAGC
19	TGTACTACTC
20	ACGACTACAG
21	CGTAGACTAG
22	TACGAGTATG
23	TACTCTCGTG
24	ACGCGAGTAT
25	ACTACTATGT
26	ACTGTACAGT
27	AGTACGCTAT
28	ATAGAGTACT
29	TACGAGTATG
30	TACTCTCGTG
31	TAGAGACGAG
32	TCGTCGCTCG
33	ACATACGCGT
34	CGTCTAGTAC
35	TCTACGTAGC
36	TGTACTACTC
37	ACGACTACAG
38	CGTAGACTAG
