# Supplementary material for: Global transcription of CRISPR loci in the human oral cavity
Source: BMC Genomics. 2015 May 21;16(1):401. doi: 10.1186/s12864-015-1615-0 (PMC4438527; doi:10.1186/s12864-015-1615-0)
Supplement: Additional file 2: Figure S1. — Plots of CRISPR spacer comparisons for each CRISPR spacer type. The Y-axis represents the number of CRISPR spacer comparisons performed and the X-axis represents the number of trinucleotide differences. The dashed line corresponds to the statistical cutoff for binning CRISPR spacers into spacer groups. Panel A—SGI CRISPR spacers, Panel B—SGII CRISPR spacers, Panel C—GHI CRISPR spacers, and Panel D—VSI CRISPR spacers. Figure S2. Rarefaction analysis of CRISPR spacer groups in the saliva of all subjects. Rarefaction curves were created using 10,000 random iterations based on spacer group richness. The y-axis represents the number of unique spacer groups and the x-axis represents the number of spacers sampled. Panel A—SGI CRISPRs, Panel B—SGII CRISPRs, Panel C—GHI CRISPRs, and Panel D—VSI CRISPRs. Periodontally healthy subjects are shown in white and subjects with significant periodontal disease are shown in black. Triangles represent CRISPR spacers from cDNA and circles represent CRISPR spacers from genomic DNA. Figure S3. Estimated percentages (± standard deviation) of CRISPR spacers shared between genomic DNA and cDNA. Within subject comparisons are demonstrated by black bars and between subject comparisons are demonstrated by the white bars. Panel A—SGI CRISPR spacers, Panel B—SGII CRISPR spacers, Panel C—GHI CRISPR spacers, and Panel D—VSI CRISPR spacers. Figure S4. Heatmaps of SGI (Panel A) and SGII (Panel B) CRISPR spacers that match virome reads. Virome reads from subjects with relative periodontal health and those with periodontal disease are shown along the rows. The columns represent the different subjects with periodontal health or disease. Figure S5. Principal coordinates analysis of beta diversity between subjects with CRISPR spacer matches to virome reads. Subjects with relative periodontal health are shown by white circles and subjects with periodontal disease are shown by black circles. Panel A—SGI CRISPRs, Panel B—SGII CRISPRs, Panel C—GHI CRISPRs, an [file 12864_2015_1615_MOESM2_ESM.pdf]

# Supplemental Figure 1

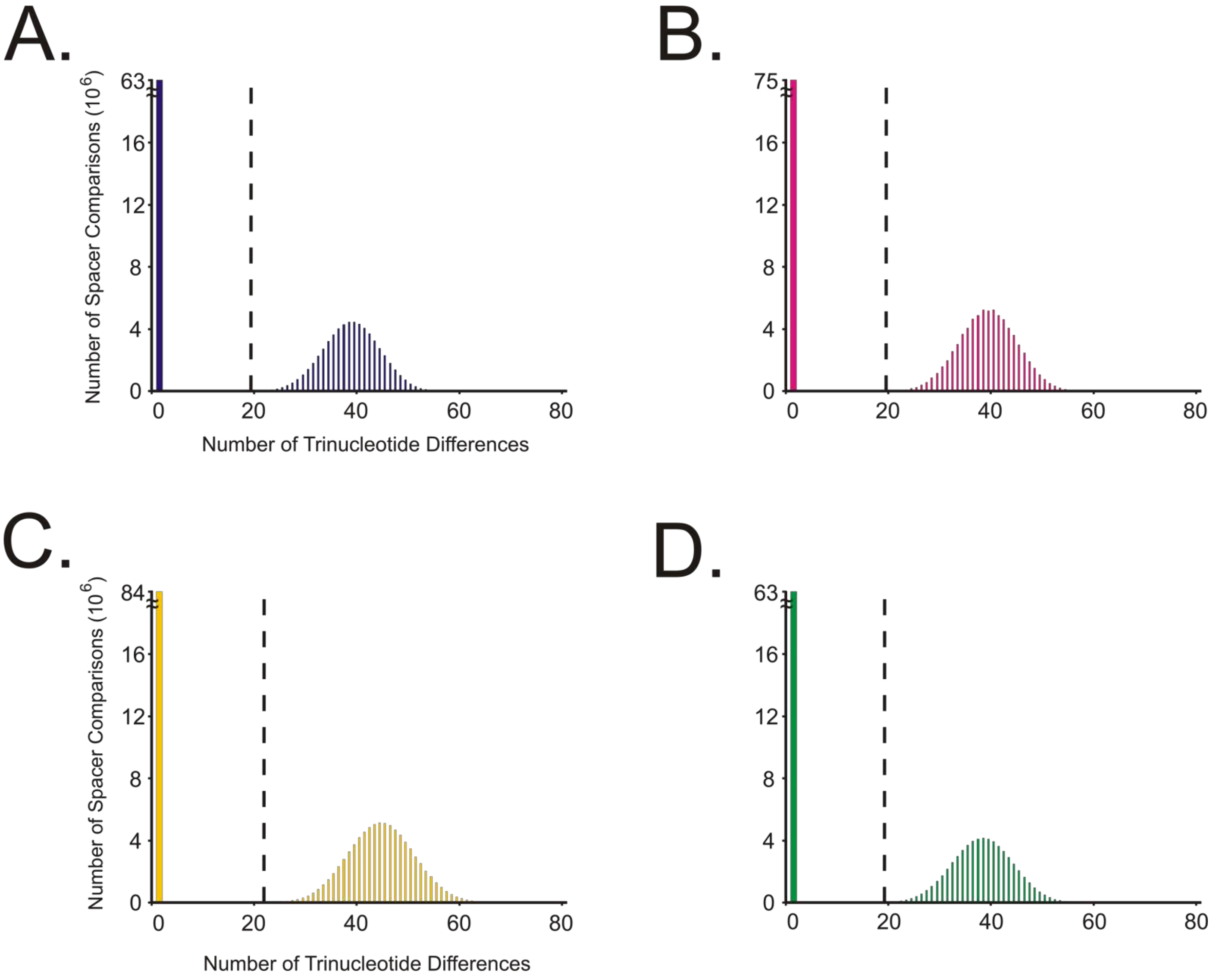


**Supplemental Figure 1**: Plots of CRISPR spacer comparisons for each CRISPR spacer type. The Y-axis represents the number of CRISPR spacer comparisons performed and the X-axis represents the number of trinucleotide differences. The dashed line corresponds to the statistical cutoff for binning CRISPR spacers into spacer groups. Panel A – SGI CRISPR spacers, Panel B – SGII CRISPR spacers, Panel C – GHI CRISPR spacers, and Panel D – VSI CRISPR spacers.

# Supplemental Figure 2

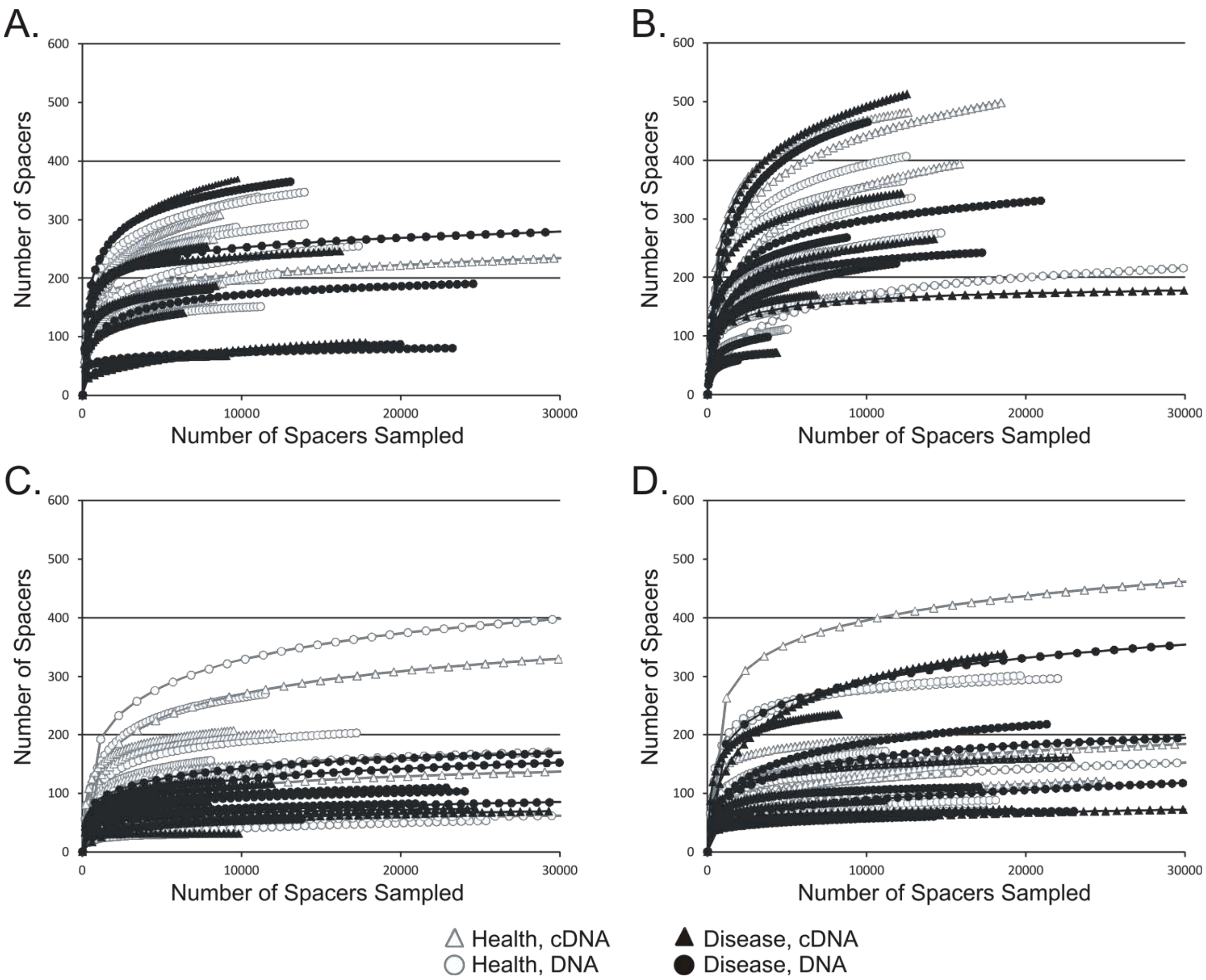


**Supplemental Figure 2**: Rarefaction analysis of CRISPR spacer groups in the saliva of all subjects. Rarefaction curves were created using 10,000 random iterations based on spacer group richness. The y-axis represents the number of unique spacer groups and the x-axis represents the number of spacers sampled. Panel A – SGI CRISPRs, Panel B – SGII CRISPRs, Panel C – GHI CRISPRs, and Panel D – VSI CRISPRs. Periodontally healthy subjects are shown in white and subjects with significant periodontal disease are shown in black. Triangles represent CRISPR spacers from cDNA and circles represent CRISPR spacers from genomic DNA.

# Supplemental Figure 3

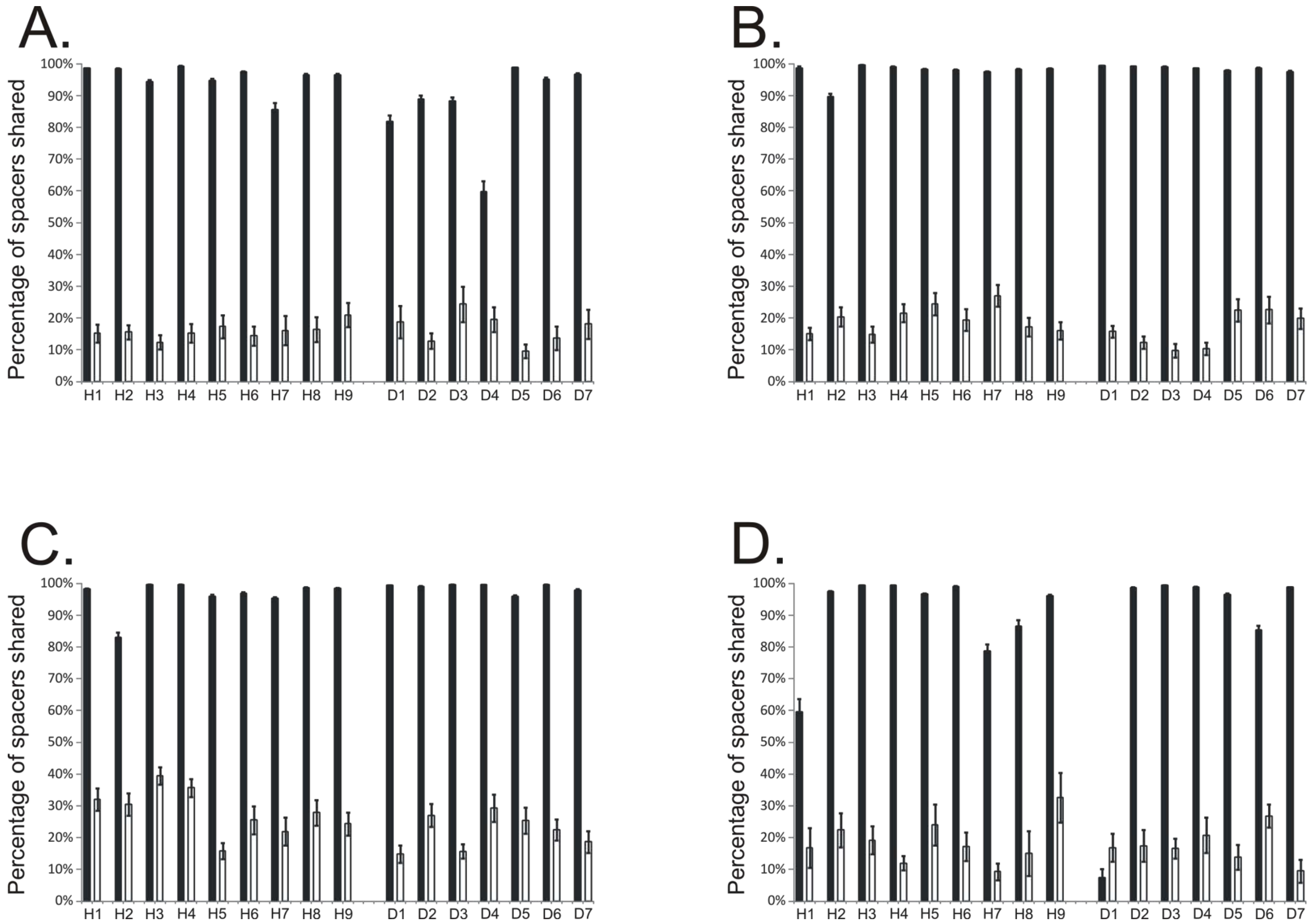


**Supplemental Figure 3:** Estimated percentages (± standard deviation) of CRISPR spacers shared between genomic DNA and cDNA. Within subject comparisons are demonstrated by black bars and between subject comparisons are demonstrated by the white bars. Panel A – SGI CRISPR spacers, Panel B – SGII CRISPR spacers, Panel C – GHI CRISPR spacers, and Panel D – VSI CRISPR spacers.

# Supplemental Figure 4

**Supplemental Figure 4**: Heatmaps of SGI (Panel A) and SGII (Panel B) CRISPR spacers that match virome reads. Virome reads from subjects with relative periodontal health and those with periodontal disease are shown along the rows. The columns represent the different subjects with periodontal health or disease.

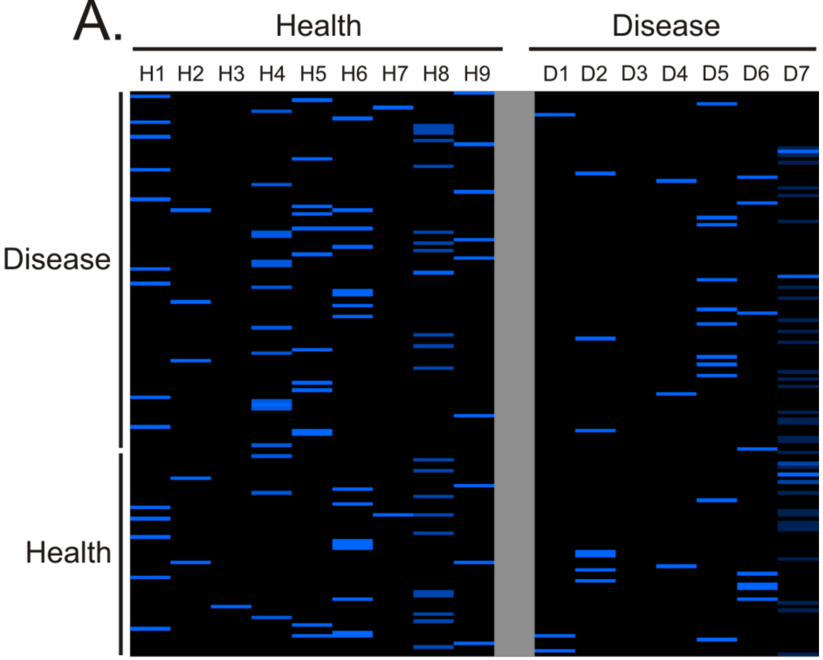


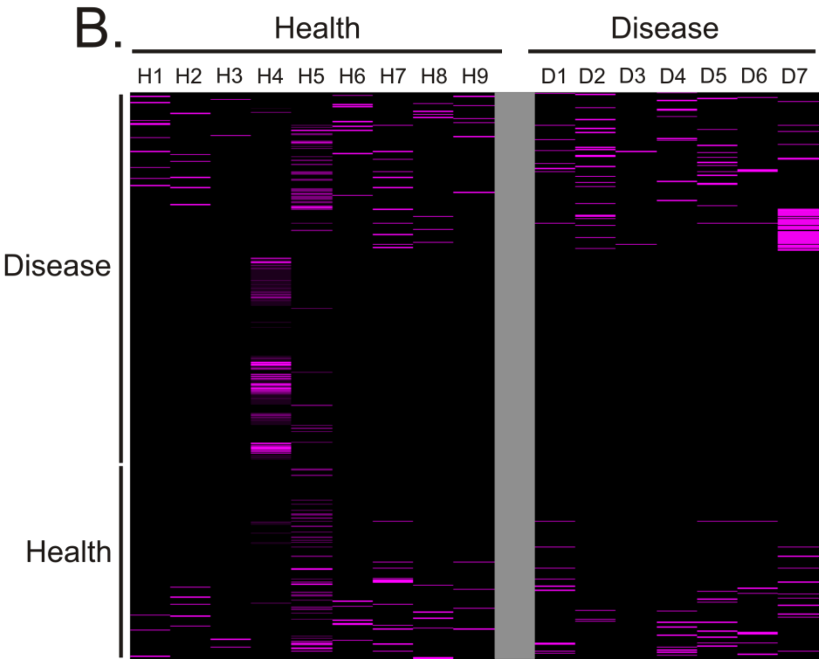

# Supplemental Figure 5

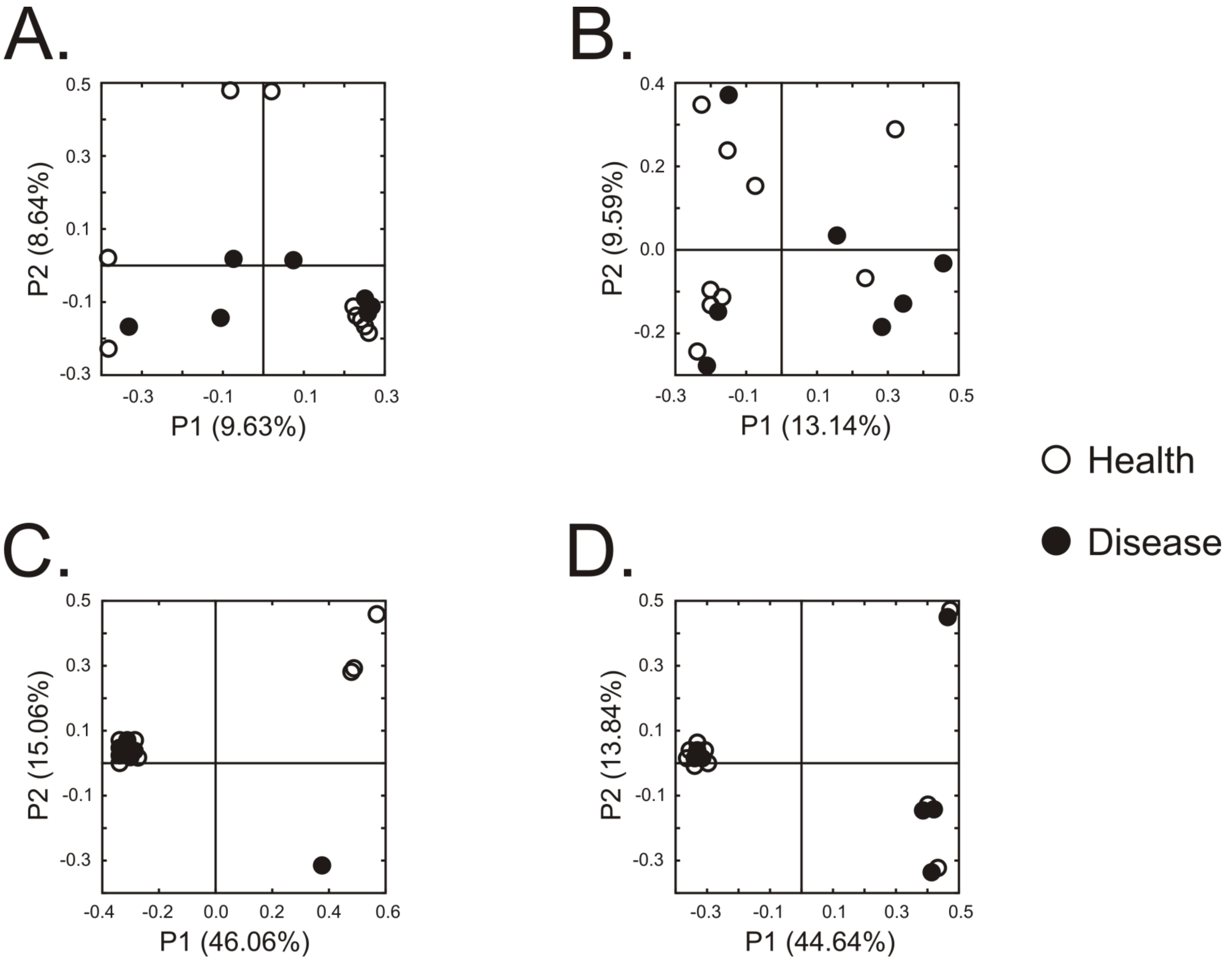


**Supplemental Figure 5**: Principal coordinates analysis of beta diversity between subjects with CRISPR spacer matches to virome reads. Subjects with relative periodontal health are shown by white circles and subjects with periodontal disease are shown by black circles. Panel A – SGI CRISPRs, Panel B – SGII CRISPRs, Panel C – GHI CRISPRs, and Panel D – VSI CRISPRs.

# Supplemental Figure 6

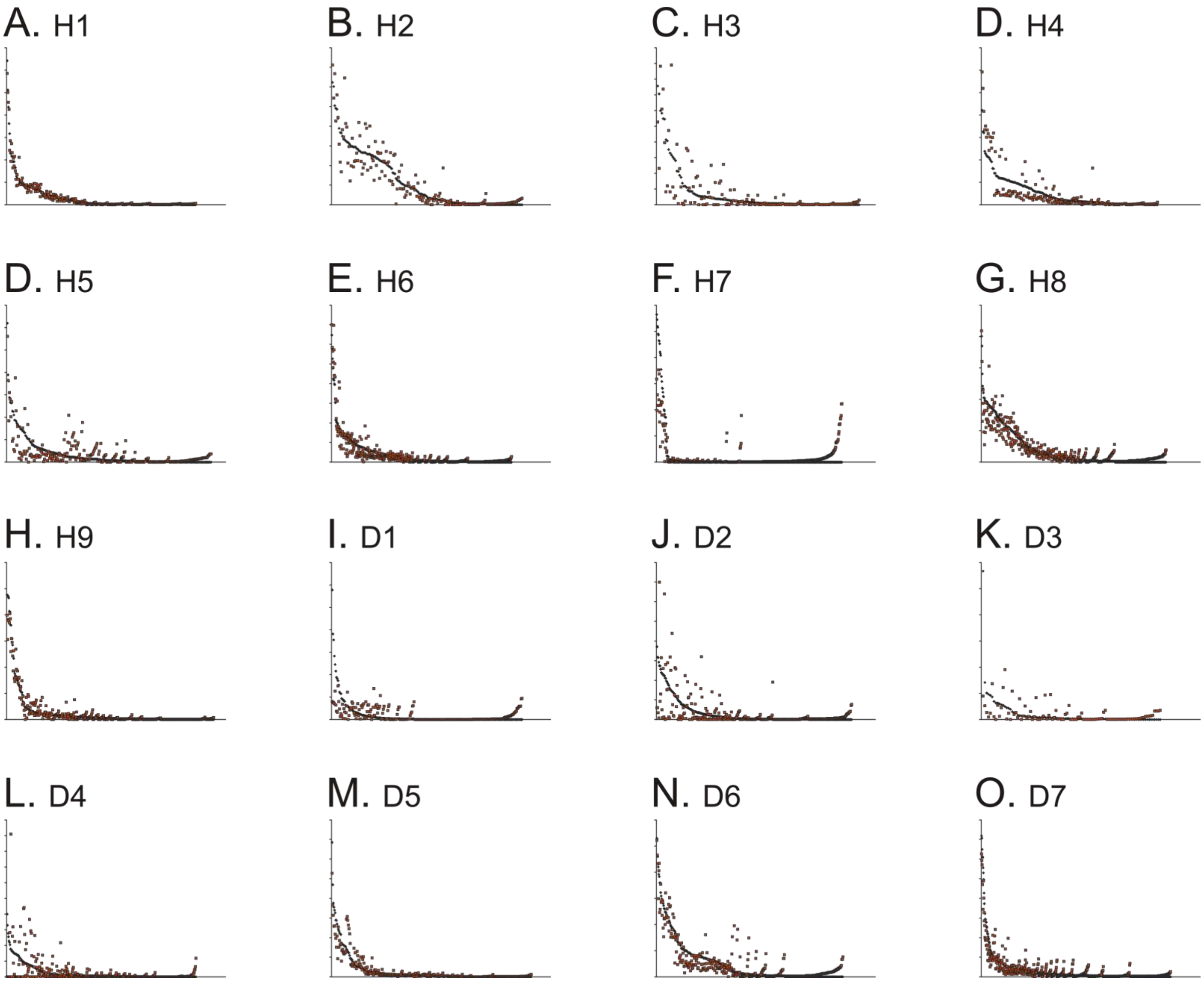


**Supplemental Figure 6**: Plots of PPTS (Percentage Per Thousand Spacers) values for SGI spacers from the DNA fractions (black diamonds) and the cDNA fractions (red boxes). The PPTS values are shown on the y-axis and the individual spacers are shown on the x-axis. Each box or diamond represents a different individual spacer. Panel A-H represent subjects with relative periodontal health and Panels I-O represent subjects with periodontal disease.

# Supplemental Figure 7

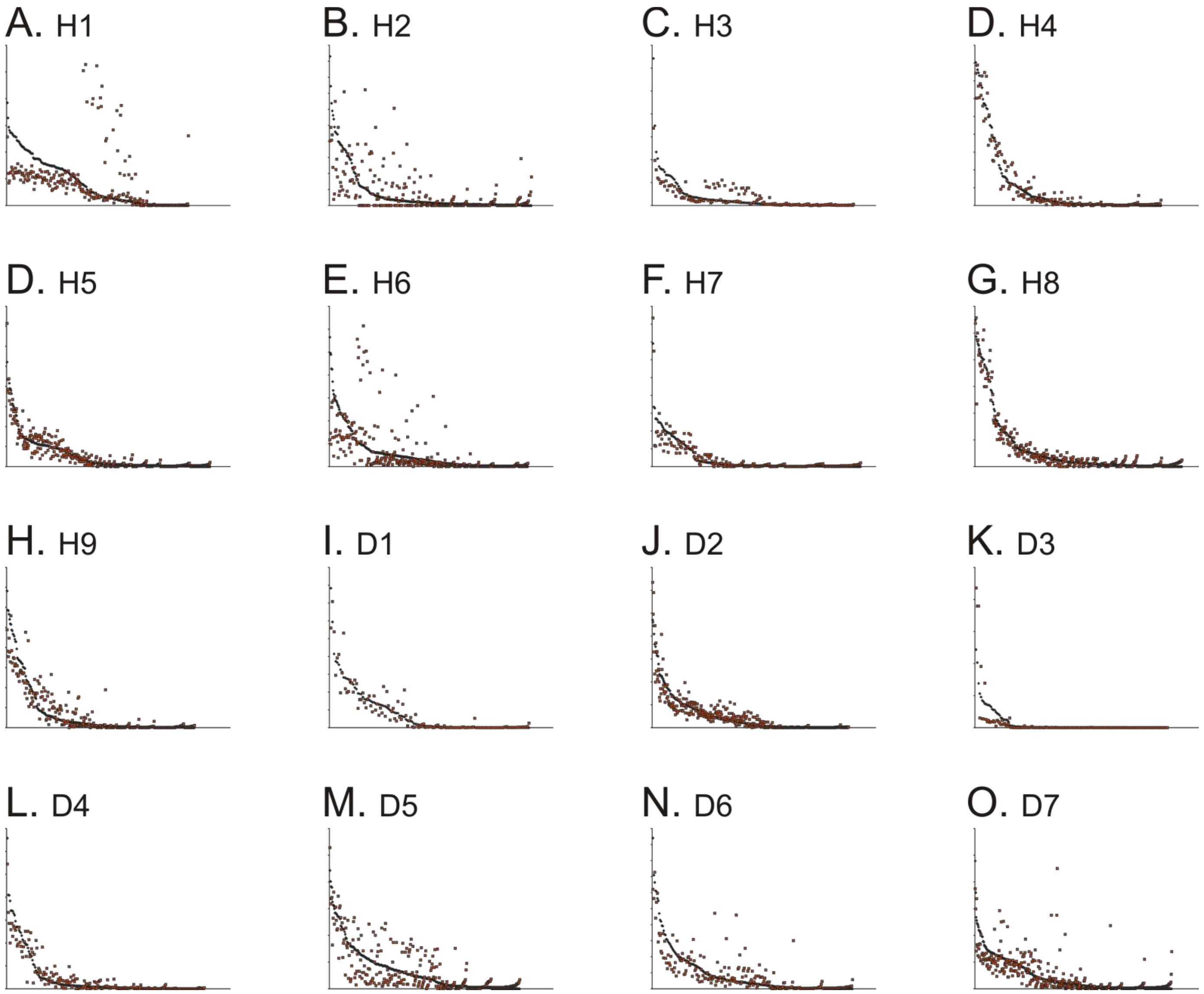


**Supplemental Figure 7**: Plots of PPTS (Percentage Per Thousand Spacers) values for SGII spacers from the DNA fractions (black diamonds) and the cDNA fractions (red boxes). The PPTS values are shown on the y-axis and the individual spacers are shown on the x-axis. Each box or diamond represents a different individual spacer. Panel A-H represent subjects with relative periodontal health and Panels I-O represent subjects with periodontal disease.

# Supplemental Figure 8

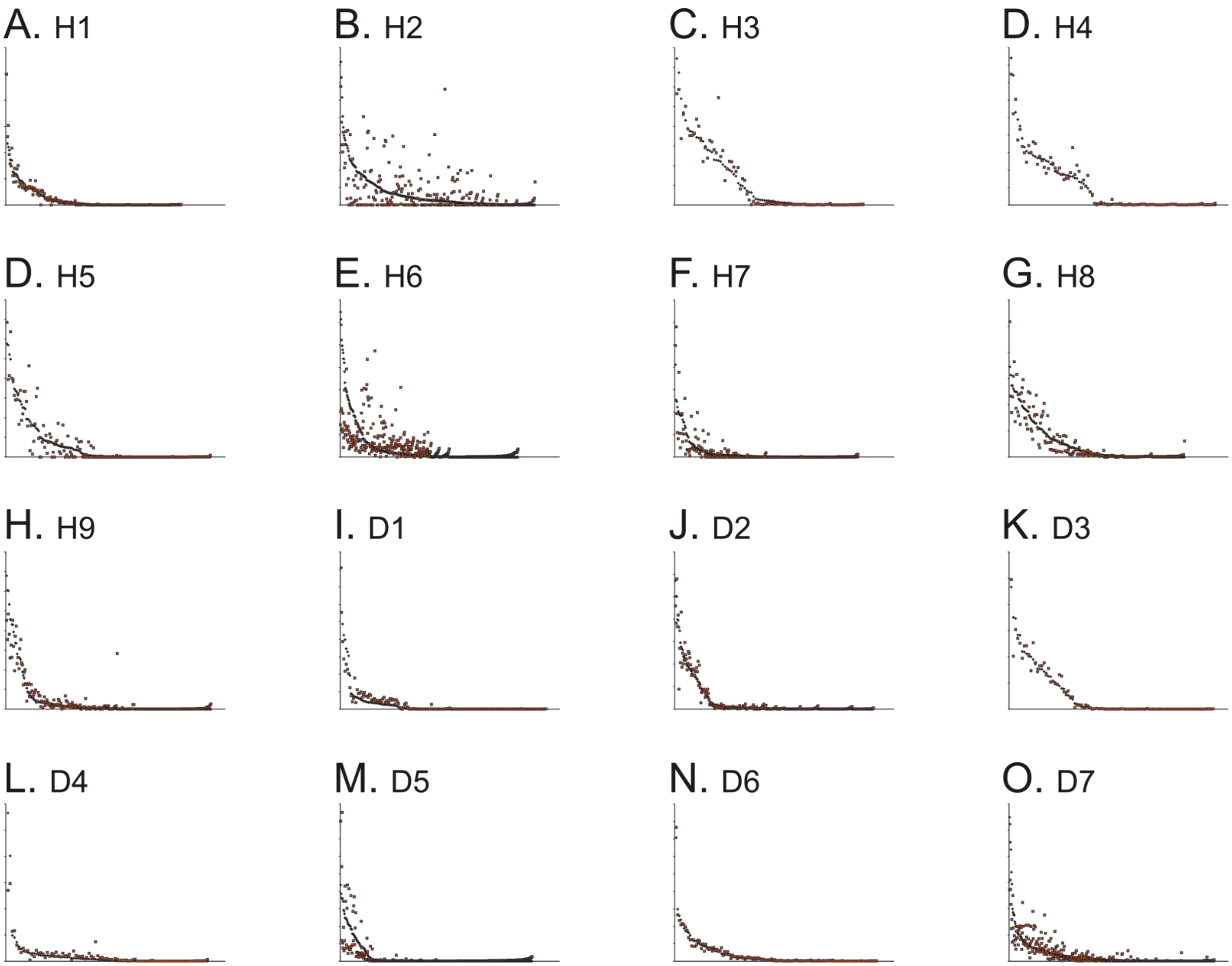


**Supplemental Figure 8**: Plots of PPTS (Percentage Per Thousand Spacers) values for GHI spacers from the DNA fractions (black diamonds) and the cDNA fractions (red boxes). The PPTS values are shown on the y-axis and the individual spacers are shown on the x-axis. Each box or diamond represents a different individual spacer. Panel A-H represent subjects with relative periodontal health and Panels I-O represent subjects with periodontal disease.

# Supplemental Figure 9

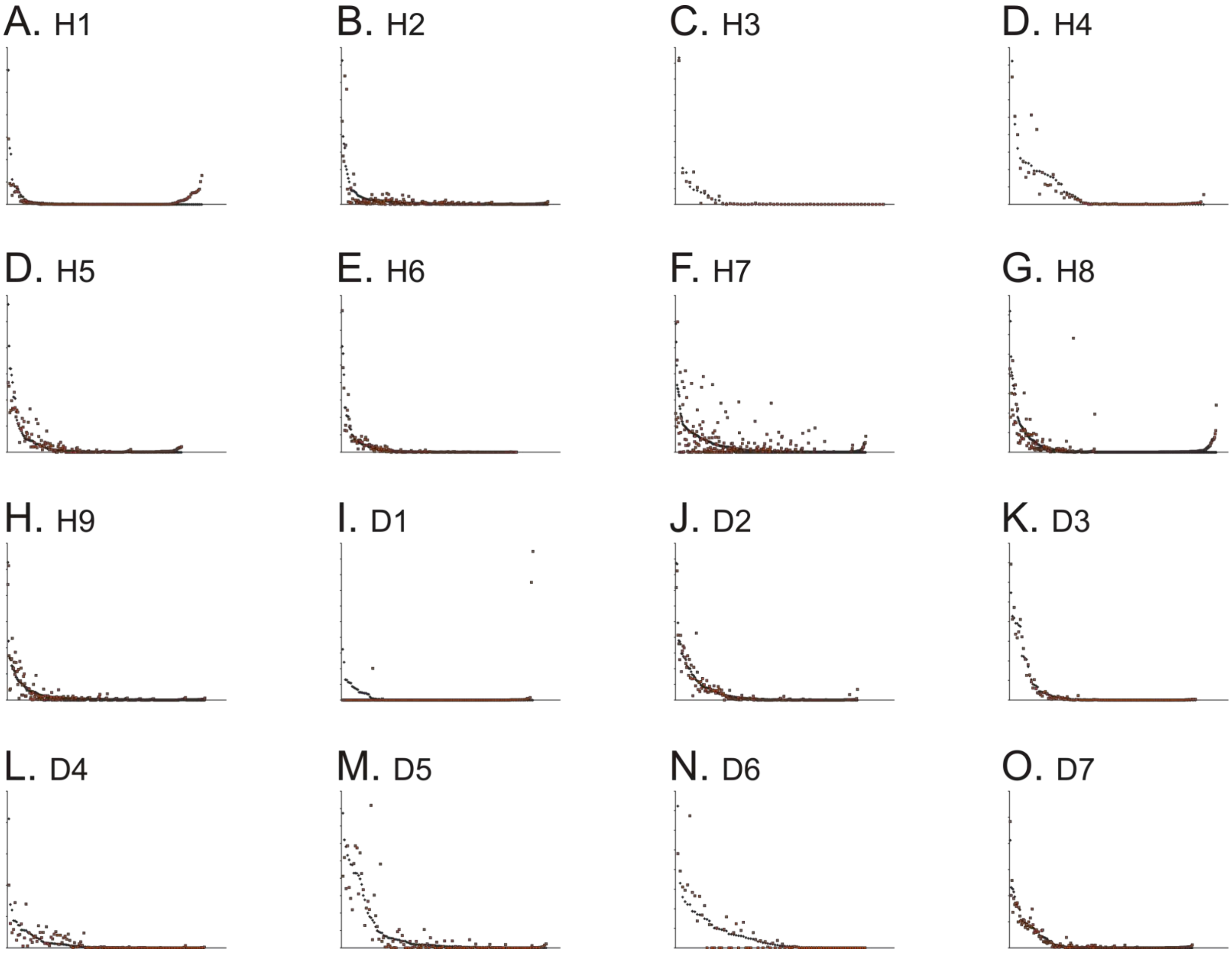


**Supplemental Figure 9**: Plots of PPTS (Percentage Per Thousand Spacers) values for VSI spacers from the DNA fractions (black diamonds) and the cDNA fractions (red boxes). The PPTS values are shown on the y-axis and the individual spacers are shown on the x-axis. Each box or diamond represents a different individual spacer. Panel A-H represent subjects with relative periodontal health and Panels I-O represent subjects with periodontal disease.

# Supplemental Figure 10

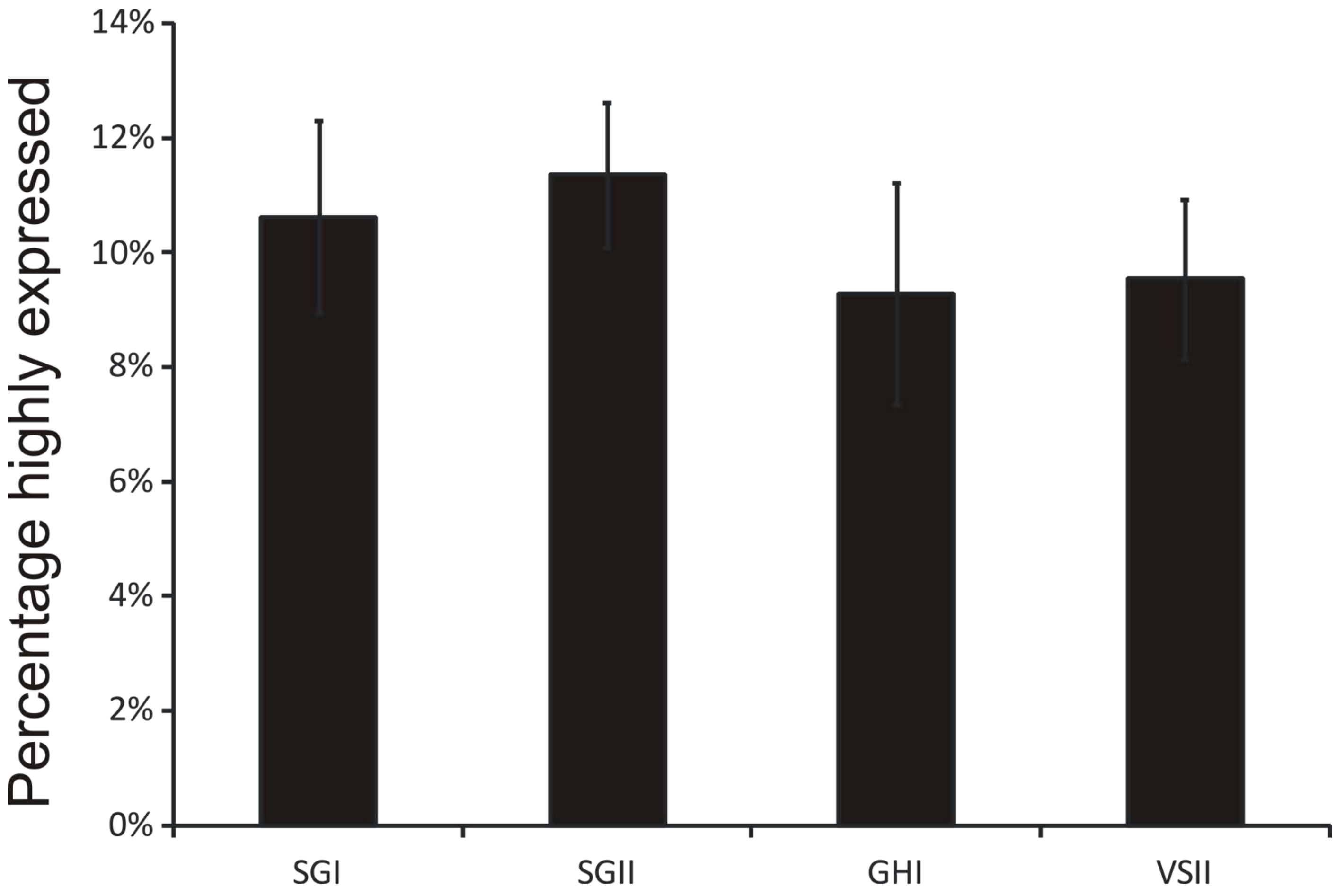


**Supplemental Figure 10**: Bar plots of the percentages (± standard deviation) of CRISPR spacers that are highly expressed. The x-axis represents the CRISPR spacer type and the y-axis represents the percentage of all spacers.

# Supplemental Figure 11

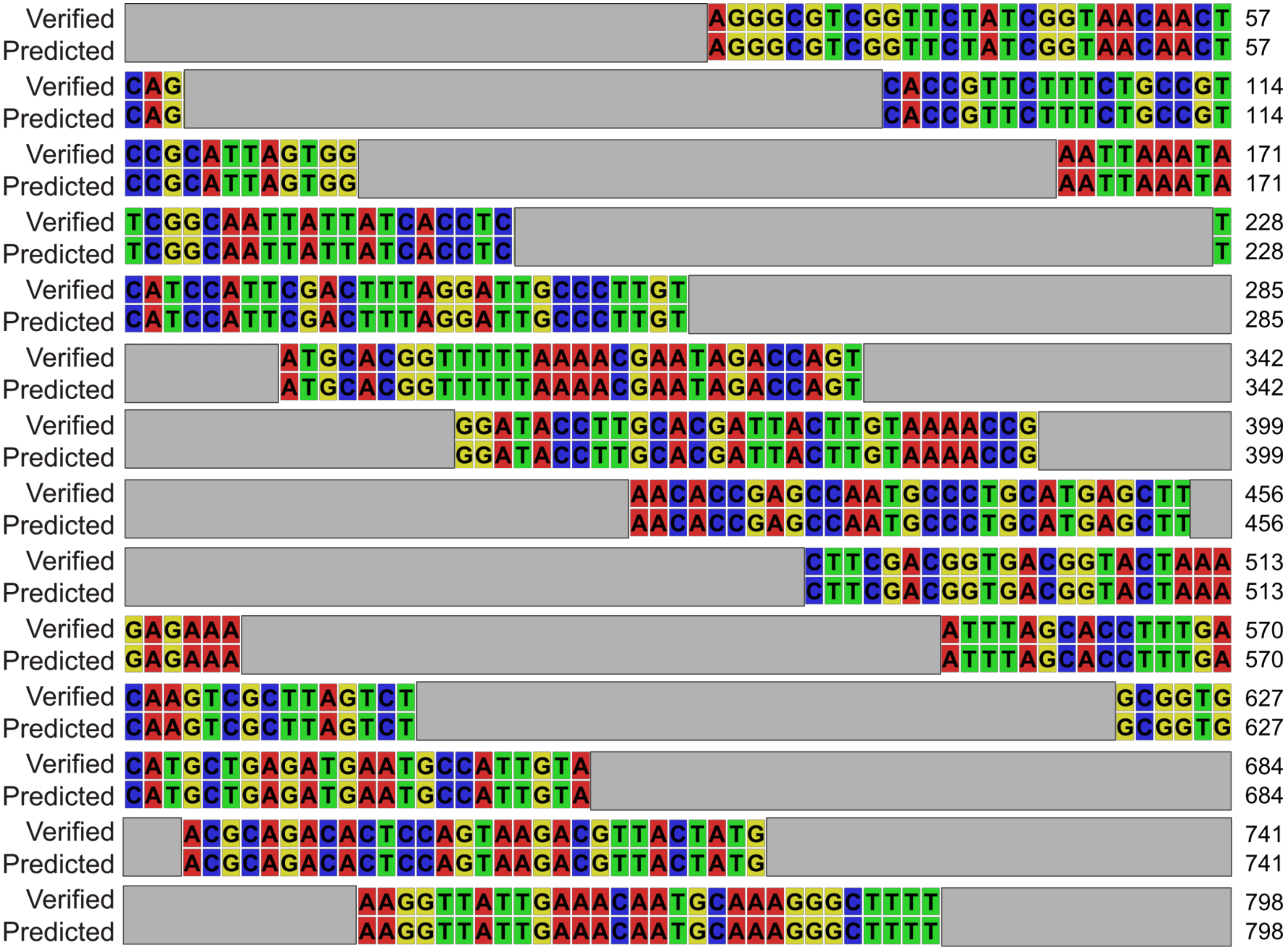


**Supplemental Figure 11**: Sequence of reassembled CRISPR locus from healthy subject H5. The 'predicted' locus represents the locus reconstructed from short read data and the 'verified' represents the locus sequence produced by Sanger sequencing of an entire amplicon. The locations of the repeat motifs are shown in grey. Nucleotide residues are colored differently for ease of demonstrating identity between the predicted and the verified locus.

# Supplemental Figure 12

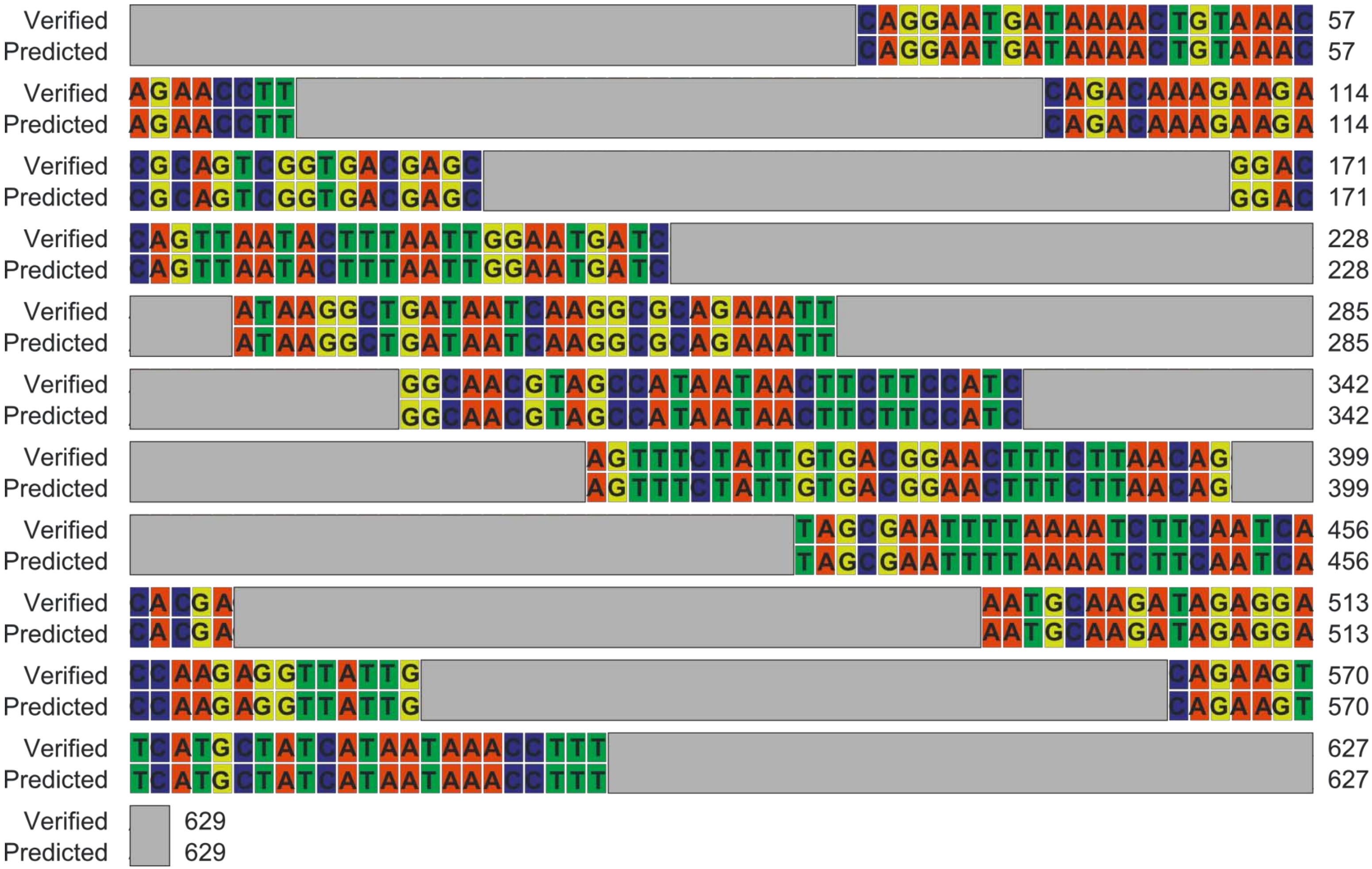


**Supplemental Figure 12**: Sequence of reassembled CRISPR locus from subject D1 with periodontal disease. The 'predicted' locus represents the locus reconstructed from short read data and the 'verified' represents the locus sequence produced by Sanger sequencing of an entire amplicon. The locations of the repeat motifs are shown in grey. Nucleotide residues are colored differently for ease of demonstrating identity between the predicted and the verified locus.

# Supplemental Figure 13

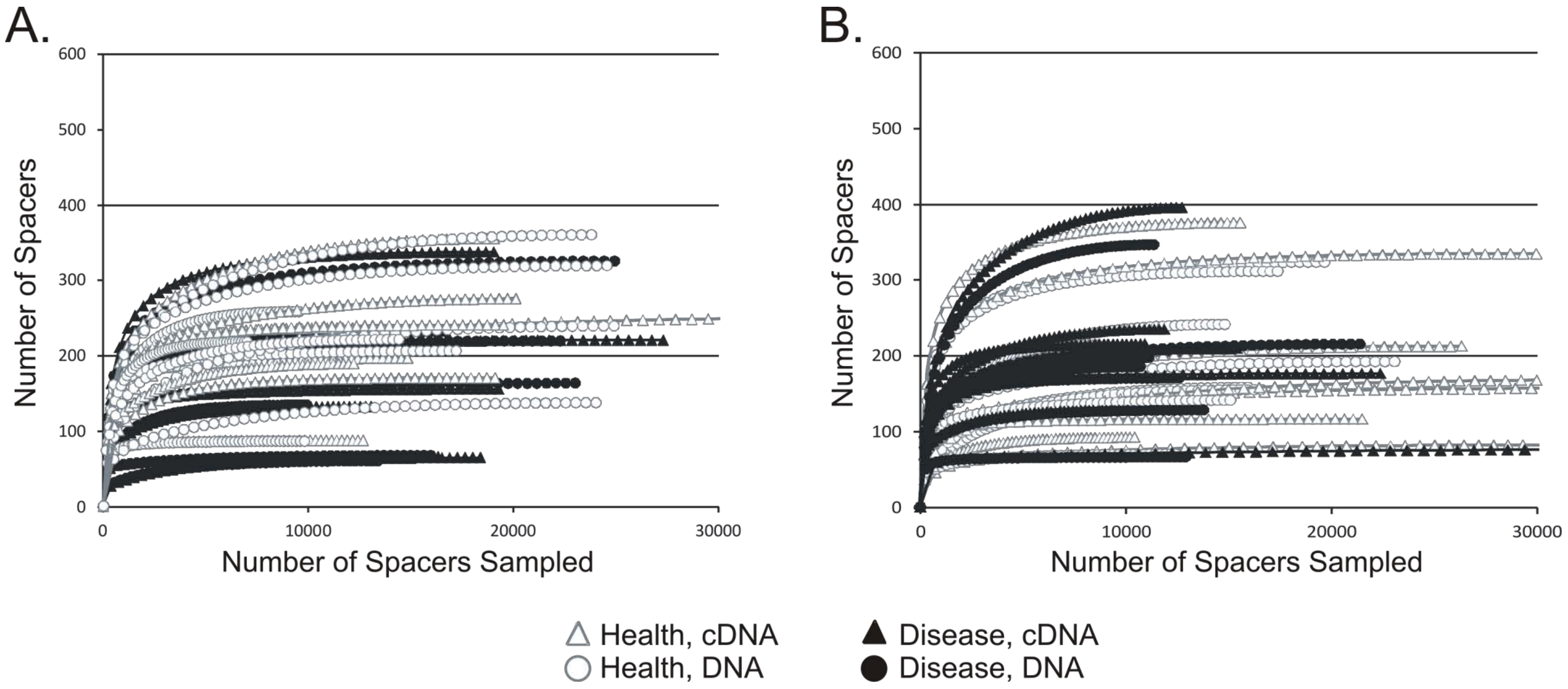


**Supplemental Figure 13**: Rarefaction analysis of CRISPR spacer groups from 200bp reads in the saliva of all subjects. Rarefaction curves were created using 10,000 random iterations based on spacer group richness. The y-axis represents the number of unique spacer groups and the x-axis represents the number of spacers sampled. Panel A – SGI CRISPRs, and Panel B – SGII CRISPRs. Periodontally healthy subjects are shown in white and subjects with significant periodontal disease are shown in black. Triangles represent CRISPR spacers from cDNA and circles represent CRISPR spacers from genomic DNA.

# Supplemental Figure 14

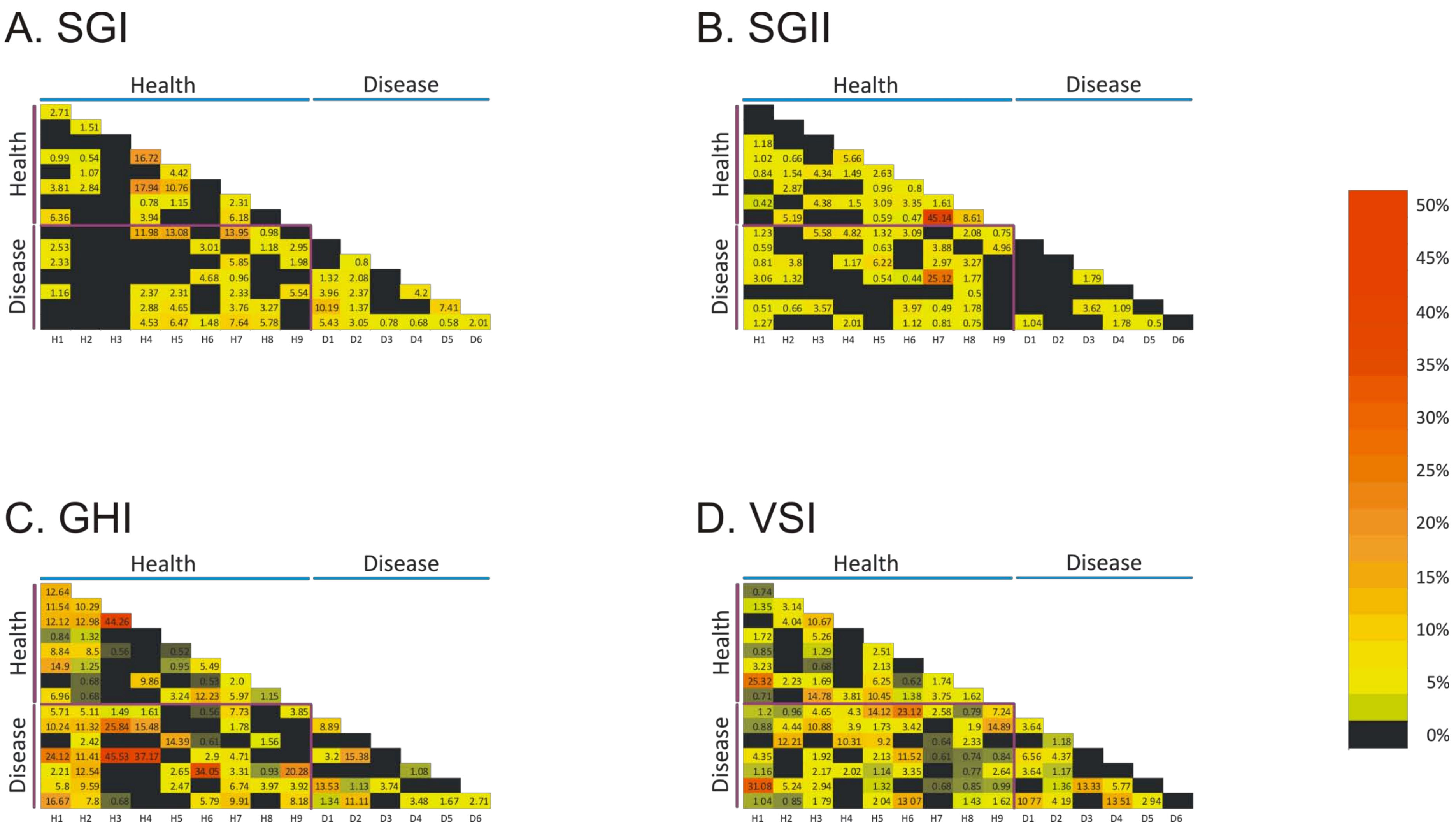


**Supplemental Figure 14**: CRISPR spacer group heat matrices demonstrating the percentage of shared CRISPR spacer groups between all subjects. The top triangular portion of each matrix represents comparisons between CRISPR spacers derived from subjects with relative periodontal health, the bottom rectangular portion of each matrix represents comparisons between periodontal health-derived and periodontal disease-derived CRISPR spacers, and the bottom triangular portion of each matrix represents comparisons between periodontal disease-derived CRISPR spacers. The intensity scale bar is located to the right. Panel A – SGI, Panel B – SGII, Panel C – GHI, and Panel D – VSI CRISPR spacers.

# Supplemental Figure 15

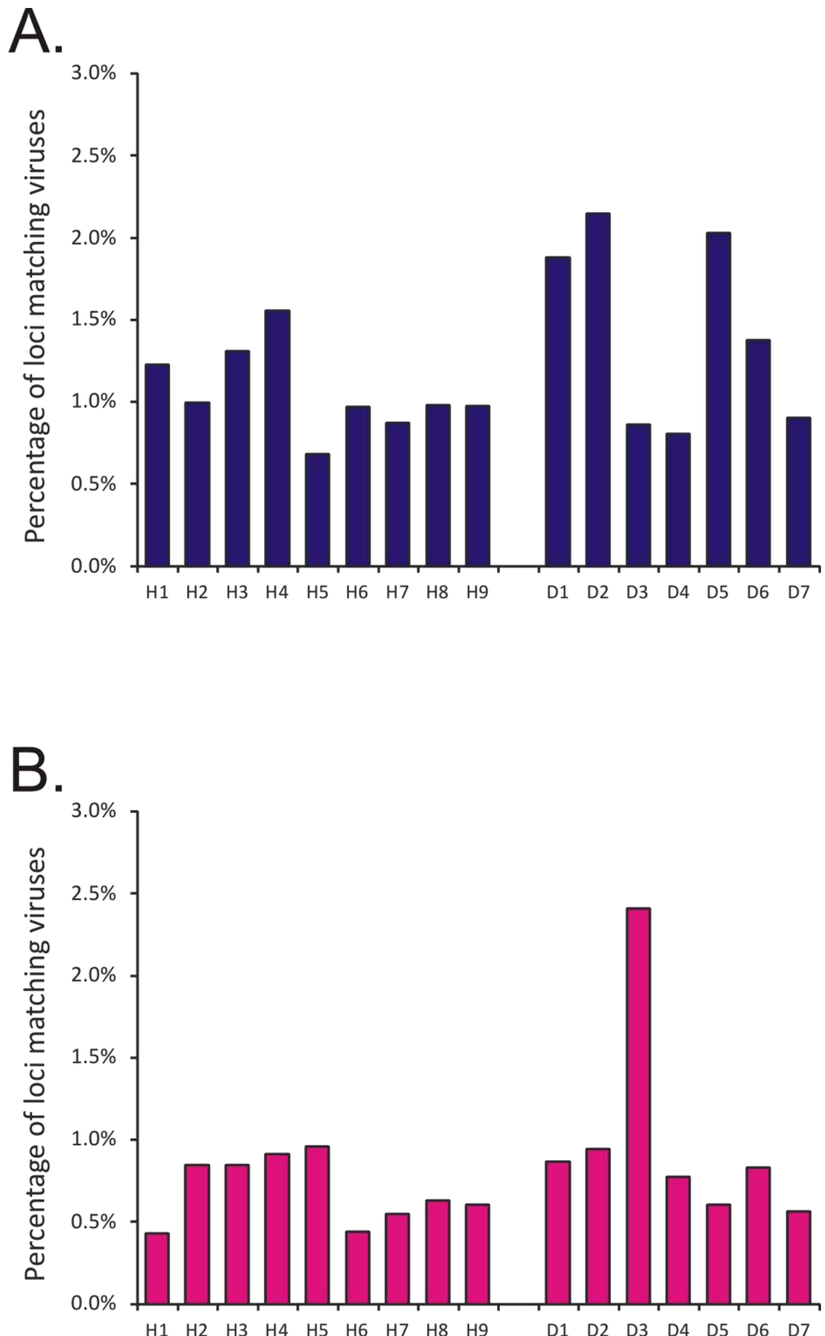


**Supplemental Figure 15**: Percentage of reassembled CRISPR loci with spacers that match virome reads for subjects with periodontal health and disease. Panel A – SGII CRISPR loci, and Panel B – SGI CRISPR loci.

# Supplemental Figure 16

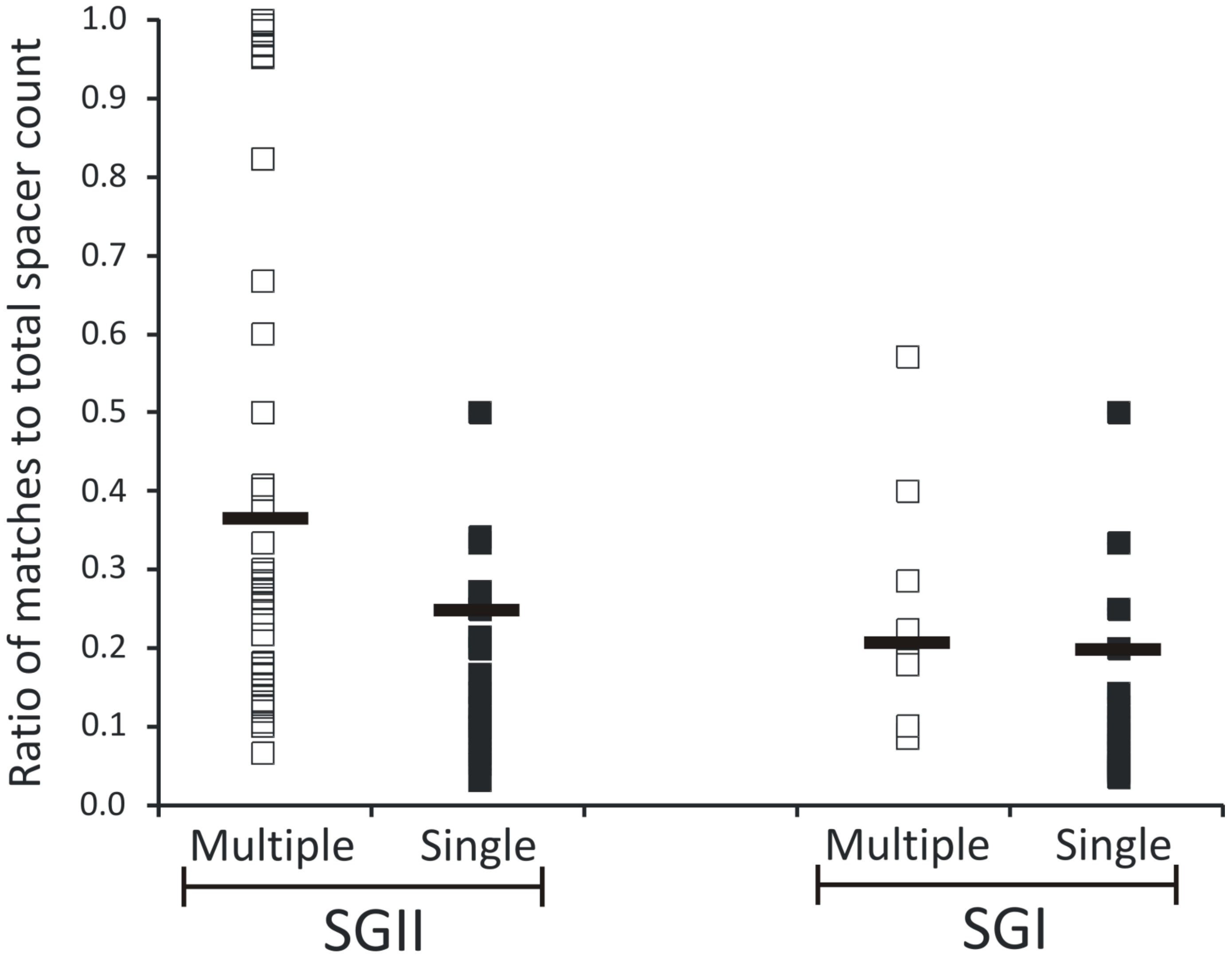


**Supplemental Figure 16**: Ratio of CRISPR spacer matches to total spacer counts in reassembled loci. The ratio of matches to spacer counts is shown for loci with multiple spacers that match viromes (white boxes) and for those with only single spacers that match virome reads (black boxes). The mean ratio for all loci from all subjects is indicated by the black bar. SGII CRISPR loci are located on the left, and SGI loci are located on the right.
